# Parsing the roles of DExD-box proteins DDX39A and DDX39B in alternative RNA splicing

**DOI:** 10.1093/nar/gkae431

**Published:** 2024-05-27

**Authors:** Shefali Banerjee, Chloe K Nagasawa, Steven G Widen, Mariano A Garcia-Blanco

**Affiliations:** Department of Microbiology, Immunology and Cancer Biology, University of Virginia, Charlottesville, VA 22903, USA; Department of Biochemistry and Molecular Biology, University of Texas Medical Branch, Galveston, TX 77550, USA; Department of Microbiology, Immunology and Cancer Biology, University of Virginia, Charlottesville, VA 22903, USA; Department of Biochemistry and Molecular Biology, University of Texas Medical Branch, Galveston, TX 77550, USA; Human Pathophysiology and Translational Medicine Program, Institute for Translational Sciences, University of Texas Medical Branch, Galveston, TX 77555-5302, USA; Department of Biochemistry and Molecular Biology, University of Texas Medical Branch, Galveston, TX 77550, USA; Department of Microbiology, Immunology and Cancer Biology, University of Virginia, Charlottesville, VA 22903, USA; Department of Biochemistry and Molecular Biology, University of Texas Medical Branch, Galveston, TX 77550, USA

## Abstract

DExD-box RNA proteins DDX39A and DDX39B are highly homologous paralogs that are conserved in vertebrates. They are required for energy-driven reactions involved in RNA processing. Although we have some understanding of how their functions overlap in RNA nuclear export, our knowledge of whether or not these proteins have specific or redundant functions in RNA splicing is limited. Our previous work has shown that DDX39B is responsible for regulating the splicing of important immune transcripts *IL7R* and *FOXP3*. In this study, we aimed to investigate whether DDX39A, a highly homologous paralog of DDX39B, plays a similar role in regulating alternative RNA splicing. We find that DDX39A and DDX39B have significant redundancy in their gene targets, but there are targets that uniquely require one or the other paralog. For instance, DDX39A is incapable of complementing defective splicing of *IL7R* exon 6 when DDX39B is depleted. This exon and other cassette exons that specifically depend on DDX39B have U-poor/C-rich polypyrimidine tracts in the upstream intron and this variant polypyrimidine tract is required for DDX39B dependency. This study provides evidence that despite a high degree of functional redundancy, DDX39A and DDX39B are selectively required for the splicing of specific pre-mRNAs.

## Graphical Abstract

**Graphical Abstract:**
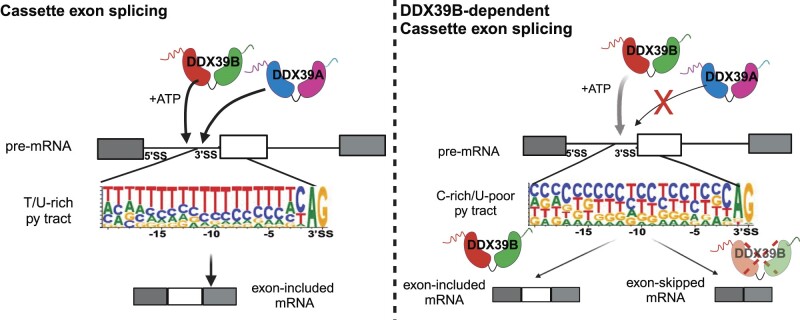

## Introduction

DExD proteins are RNA-dependent ATPases, which are a sub-family of the helicase superfamily 2 (SF2) and are involved in various aspects of RNA metabolism. All DExD-box proteins consist of a structurally conserved helicase core composed of the sequence motifs required for ATP-binding, and ATPase and helicase activities. The sequence motifs that surround the conserved helicase core differ among DExD-box proteins and are responsible for their distinct functions (1,2). DExD-box proteins act as energy-driven motors to facilitate productive interactions between RNA–RNA and RNA–protein in large RNP complexes, such as those involved in RNA transcription, splicing, translation, and mRNA decay. The highly homologous DDX39A and DDX39B proteins (>90% sequence identity; Supplementary Figure S1), which are the vertebrate orthologs of *S.cerevisiae* Sub2, are required for RNA export and splicing. DDX39A and DDX39B can both compensate for the lethal loss of Sub2 in yeast, implying that the DDX39 proteins have evolutionarily conserved roles in RNA processing (3). Notably, a study previously showed that DDX39A controls the nucleocytoplasmic export of short circular RNAs (circRNAs), whereas DDX39B is required for the nuclear export of long circRNAs in human cells (4). Recent work from the Masuda group indicates that DDX39A and DDX39B can form different nuclear export complexes in the absence of ATP (5–7). These studies provide some evidence of factors that modulate DDX39A and DDX39B function, giving them distinct roles in the nuclear export of RNAs. Our understanding of the specific role(s) that DDX39A or DDX39B play in RNA splicing is less well-understood.

Pre-mRNA splicing is catalyzed by the spliceosome machinery, formed by the stepwise assembly of four small ribonucleoprotein complexes (U1, U2, U5, U4/U6) and a large number of splicing factors. Spliceosome formation is a dynamic process that requires ATP-hydrolysis. DDX39B, aka UAP56, is an essential splicing factor required for at least two early steps in the spliceosome assembly(8–10). DDX39B is recruited to pre-mRNA by U2AF2 in an ATP-dependent manner. The ATPase activity of DDX39B is required to displace SF1 from the branchpoint sequence (BPS) and recruit U2snRNP to the BPS, forming the pre-spliceosome (aka complex A) (10). DDX39B also interacts with U1snRNA stem-loop III in an ATP-dependent manner, which enhances the contact between U1 and U2 snRNPs on the pre-mRNA (11). A study found that DDX39B interacts with U4 and U6 snRNAs and helps in the unwinding of U4/U6 in a U2AF65-dependent manner (8). These experiments do not show that this unwinding happens in the context of spliceosome formation, which is well established for Brr2 (12–14) and are thus not definitive. Nonetheless, the data suggest that DDX39B plays a role in spliceosome assembly after formation of the pre-spliceosome (8).

We and others have shown that DDX39B plays a role in alternative splicing (15–17). DDX39B activates *IL7R* exon 6 splicing and promotes the production of the full-length IL7R receptor instead of soluble form of the receptor (sIL7R). High levels of sIL7R are associated with a high risk of developing multiple sclerosis (MS)(18), and there is genetic and functional epistasis for MS risk between these *IL7R* alleles and alleles in *DDX39B* (15) In a recent study, we established that DDX39B activates *FOXP3* intron splicing and regulates the expression of FOXP3(16), a lineage-defining transcription factor that is essential for T regulatory cell function (19–21). These findings indicate critical roles for DDX39B activity in regulating immune function. Our previous studies suggested, but did not formally establish, that the regulation of these splicing decisions with important consequences for immunity were not affected by DDX39A. This open question led us to determine the extent of functional overlap between DDX39B and its closely related paralog, DDX39A.

One study had previously indicated that depleting DDX39A and DDX39B results in decreased expression of alternatively spliced variants of the androgen receptor (AR) in prostate cancer cells (17). This study, however, did not address whether the effect of DDX39A and DDX39B depletion on AR splice variant expression is mediated through the regulation of RNA splicing or nuclear export. To our knowledge, Martelly *et al.*, is the only study that has unambiguously shown the involvement of both DDX39A and DDX39B in spliceosome assembly. They show that both DDX39A and DDX39B proteins can interact with U1 snRNA stem-loop III and promote further interactions between U1 and U2 snRNPs (9). Most previous studies investigating the role of DDX39 proteins in RNA splicing, including our own work, have not unambiguously determined the involvement of DDX39A or DDX39B.

In this study, we investigated the overlap in function between DDX39A and DDX39B in regulating alternative RNA splicing. We find that DDX39A and DDX39B have significant redundancy in their gene targets, but there are targets that uniquely require one or the other paralog. We focused on cassette exons that were specifically sensitive to DDX39B disruption, and we observed that cassette exons with U-poor/C-rich polypyrimidine tracts in the upstream intron were sensitive to DDX39B levels and were skipped more upon depletion of DDX39B, but not DDX39A. We also confirmed our previous work showing that introns containing U-poor/C-rich polypyrimidine tracts are sensitive to DDX39B levels (16,22), and now show that these are insensitive to DDX39A disruption. Among the introns retained more upon DDX39A or DDX39B depletion were *DDX39A* and *DDX39B* intron 6, respectively. For DDX39B dependency of both cassette exons and introns we note a special role for U-poor/C-rich polypyrimidine tracts.

## Materials and methods

### Cell lines and cell culture conditions

HeLa (ATCC CCL-2) and HeLa Flp-In-Trex (Thermo Fisher Scientific) cells were grown in DMEM medium (Gibco) supplemented with 10% (v/v) heat-inactivated fetal bovine serum and 1% (v/v) penicillin-streptomycin (Thermo Fisher Scientific). THP1 cells were obtained from Alexander Bukreyev's lab (UTMB Galveston) and were grown in RPMI medium (Gibco) supplemented with 10% (v/v) heat-inactivated fetal bovine serum. All cells were tested using the MycoAlert Mycoplasma Detection Kit (Lonza) and were confirmed to be free of Mycoplasma contamination.

### Generation of stable cell lines expressing DDX39A or DDX39B transgene

HeLa cells stably expressing DDX39B cDNA transgene were generated as previously described (15). HeLa cells stably expressing an inducible DDX39A cDNA trans-gene were generated using the Flp-In T-Rex system (Thermo Fisher Scientific) as recommended by the manufacturer. The coding sequence of DDX39A (NC_000019.10) was amplified with Phusion High-Fidelity DNA polymerase (New England BioLabs) using cDNA as the template prepared from total RNA isolated from HeLa cells. The resulting PCR amplicon was cloned into XhoI-digested pcDNA5/FRT/TO plasmid using Gibson assembly and verified by Sanger sequencing. Geneblocks for the respective DDX39A-DDX39B chimeras were used for Gibson assembly. The recombinant plasmids carrying the insert were co-transfected with pOG44 plasmid (1:9 ratio), which encodes the Flp recombinase, into HeLa Flp-In TRex cells using Lipofectamine 2000 (Thermo Fisher Scientific) according to the manufacturer's instructions. Transfected cells were grown in DMEM medium supplemented with 10% FBS free of doxycycline (GE Healthcare) under blasticidin/hygromycinB (300 μg/ml) selection for 15 days, and resistant cells were expanded and used for subsequent experiments. Expression of the transgene was induced by the addition of doxycycline at 1 μg/ml.

### RNAi-mediated knockdown of DDX39A or DDX39B

siRNA duplexes against DDX39A and DDX39B were purchased from Qiagen and diluted to a final concentration of 20μM upon arrival. Two independent siRNAs were used to account for potential off-target effects (siD06 and siD09 targeting DDX39A expression and siD11 and siD13 targeting DDX39B expression). AllStars Negative Control siRNA (Qiagen) was used as negative control in all knockdown experiments. Transfections were performed in biological triplicates for each siRNA. 5 × 10^4^ HeLa cells were seeded in 500 μl of DMEM medium per well in 24-well plate format. siRNAs were diluted in Opti-MEM I medium (Thermo Fisher Scientific) and transfected at a final concentration of 5 nM for DDX39B siRNAs and 25 nM for DDX39A siRNAs using Lipofectamine RNAiMax (Thermo Fisher Scientific), following the manufacturer's recommendations. 48 hours post-transfection, cells were harvested with Trizol Reagent (Thermo Fisher Scientific) for RNA isolation or with 1X RIPA buffer for protein extraction. Knockdown efficiency was estimated by western blot analysis, RT-qPCR or mass spectrometry. It was difficult to accurately measure DDX39A protein expression solely using western blot analysis because commercially available antibodies targeting DDX39A (anti-DDX39) cross-react with DDX39B.

DDX39A and DDX39B knockdown in THP1 cells was carried out using Amaxa 4D-Nucleofector (Lonza). 2 × 10^6^ cells were transfected with 30nM of each siRNA (siD09 targeting DDX39A and siD13 targeting DDX39B) using the SG Cell Line 4D-Nucleofector™ X Kit (Lonza). 48 h post nucleofection, cells were lysed using Trizol or 1× RIPA buffer for RNA isolation and protein extraction, respectively.

For the rescue experiments, HeLa cells stably expressing a DDX39A or DDX39B cDNA trans-gene were grown in the absence or the presence of doxycycline to control expression of the trans-gene. Cells were transfected with the corresponding siRNAs on the day of seeding. For rescue experiments, we used siRNAs siD09 and siD13 targeting the 3′ UTR of the endogenous DDX39A and DDX39B transcripts, respectively, which is not present in transcripts from the cDNA trans-genes. 48-hour post transfection with siRNAs, transgene expression was induced with 1 ug/ml of doxycycline. Cells were harvested on day 3 with Trizol for RNA isolation or with 1× RIPA buffer protein extraction.

### Targeted mass spectrometry analysis

Protein levels in DDX39A-knockdown (siD06 and siD09), DDX39B-knockdown (siD11 and siD13), and control (NSC-5nM and NSC-25nM) samples were determined using the Pierce BCA Protein Assay kit (Thermo Fisher Scientific). Each condition had three biological replicates. 10 μg of protein was extracted from each sample and used to create peptide samples for LCMS analysis using the S-TRAP^TM^ (Protifi) processing and trypsin digestion method. The resulting peptide samples were analyzed using the Orbitrap Fusion Mass Spectrometer (Thermo Fisher Scientific). The RAW data were processed using Proteome Discoverer with the Sequest algorithm and searched against the human database. DDX39A and DDX39B expression levels were quantified using targeted mass spectrometry with the Skyline software package. Three unique peptide pairs for DDX39A (HF**V**LDECDK, V**S**VFFGGLSIK, VNI**V**FNYDMPEDSDTYLHR) and DDX39B (HF**I**LDECDK, V**A**VFFGGLSIK, VNI**A**FNYDMPEDSDTYLHR) were identified, and the peak areas for each peptide were normalized to the corresponding NSC values. The only peptide that yielded reproducible results was DDX39A-HF**I**LDECDK and DDX39B-HF**V**LDECDK. The peak areas for siD06 and siD09 were normalized to NSC-25nM, and the peak areas for siD11 and siD13 were normalized to NSC-5nM.

### RNA Sequencing, gene expression and RNA splicing analysis

Total RNA was isolated from DDX39A-knockdown (siD06 and siD09), DDX39B-knockdown (siD11 and siD13), and control (NSC-5nM and NSC-25nM) samples using a Direct-zol RNA Purification kit (Zymoresearch). The level of knockdown in each sample was confirmed with targeted mass spec analysis (as previously explained) and RT-qPCR. Only those samples that had the lowest expression of DDX39A at the peptide level in siD06 and siD09 samples and lowest expression of DDX39B at the peptide level in siD11 and siD13 samples were selected. We sequenced two replicates each for DDX39B knockdown with siD11 and siD13. For DDX39A knockdown, we sequenced three replicates for siD09 and two replicates for siD06 conditions, respectively. Three replicates for each of the two control samples (NSC-5nM and NSC-25nM) were sequenced. Poly-A + RNA was enriched from 1 μg of total RNA and used as the template to generate paired-end libraries using the NEBNext polyA mRNA Magnetic Isolation Module and NEB Ultra II RNA library preparation kit for Illumina following manufacturer's protocol. Libraries were sequenced on a 2 × 100 paired-end format on an IlluminaNextSeq 550. Quality control of the sequenced raw data was carried out using FastQC. We obtained 70–100 million reads per sample. Reads were mapped to the human GRCh38 reference with Hisat2 (23), and read counts were calculated using Featurecounts R scripts. DEseq2 (24) was used to calculate the differential expression of genes (DEG) for each condition. The RNAseq data was deposited at Gene Expression Omnibus accession number: GSE253261.

Gene expression for the DDX39A-knockdown group was compared with the NSC-25nM control group. The DDX39B-knockdown group was compared with the NSC-5nM group for DEG analysis. The DDX39A-knockdown group consisted of two replicates each of the siD06 and siD09 conditions, and the DDX39B-knockdown group consisted of two replicates each of the siD11 and siD13 conditions. In total, there were four independent replicates for each knockdown condition. To determine the significantly altered transcripts, we used an initial cutoff of baseMean of greater than 50. We used a secondary cutoff of padj value < 0.05 and |log_2_foldchange| > 0.36 to identify the transcripts that were significantly altered between the control and knockdown conditions. The list of differentially regulated genes upon DDX39A and DDX39B knockdown are in Supplementary Files 1 & 2, respectively.

For splicing analyses, we used rMATS (25) using the default parameters. We used the same comparison strategy as previously explained for DEG analysis to determine the alternative splicing changes upon knockdown. The DDX39A knockdown group was compared to NSC-25nM, and the DDX39B knockdown group was compared to NSC-5nM. A cutoff of *P* value <0.05 and absolute value of ‘IncLevelDifference’ >0.1 was used to define alternative splicing changes that were differentially regulated upon knockdown. Reads mapped onto the UCSC Genome Browser (hg38) were used to make coverage tracks to visualize read levels for transcript abundance and alternative splicing. rMAPS analysis (25) for the U2AF2 binding motif in the introns of cassette exons included more (*n* = 285) and included less (*n* = 396) upon DDX39B knockdown was carried out using default parameters. The list of differentially regulated alternative splicing changes upon DDX39A and DDX39B knockdown are in Supplementary Files 3 & 4, respectively.

### Gene set enrichment analysis (GSEA) for DEG and RNA splicing.

To conduct GSEA analysis (26) for the differentially expressed genes upon DDX39A and DDX39B knockdown, we created gene sets of DDX39A up- and downregulated genes and gene sets of DDX39B up and downregulated genes. Genes that pass the cutoff of *P*_adj_ value < 0.05 and |log_2_fold change| > 0.36 were included in the gene sets. We generated two independent gene lists of the differentially expressed genes upon DDX39A and DDX39B knockdown. Only the genes that pass the cutoff values of baseMean > 50 and *P*_adj_ value < 0.05 were included in the gene lists.

Cassette exons with inclusion level differences <−0.1 upon DDX39A and DDX39B knockdown were used for constructing gene sets for GSEA analysis of exons skipped more upon each knockdown. Similarly, introns with inclusion level differences >0.1 upon DDX39A and DDX39B knockdown were used for constructing the gene sets for introns retained more upon each knockdown. The cassette exons lists consisted of exons differentially regulated (*P-*value < 0.05) upon DDX39A knockdown and DDX39B knockdown and the intron lists consisted of introns differentially regulated (*P-*value < 0.05) upon DDX39A and DDX39B knockdown.

### Py tract sequence analysis of skipped exons and retained introns.

Sequences of 3′ SS (from –20) were defined as polypyrimidine tract (py tract) sequence, and nucleotide probability at each position was determined per gene. The sequence logos were generated by WebLogo3. For comparison of py tracts of introns that were DDX39A-sensitive (more retained upon DDX39A knockdown) and DDX39B-sensitive (more retained upon DDX39B knockdown), we compared the 34 introns that were significantly retained upon DDX39A knockdown, and 89 introns significantly retained upon DDX39B knockdown versus 100 introns randomly selected from unchanged introns. For comparison of py tracts of cassette exons that were DDX39A-sensitive (skipped more upon DDX39A knockdown) and DDX39B-sensitive (skipped more upon DDX39B knockdown), we compared 160 skipped exons upon DDX39A knockdown, 396 skipped exons upon DDX39B knockdown versus 150 exons randomly selected from unchanged exons.

### Reporter construction and transfection

The IL7R splicing reporter, as previously described (18), consisted of the genomic region of *IL7R* (NC_000005.10) encompassing 614 bp of intron 5, exon 6, and 573 bp of intron 6, cloned in between constitutive upstream (U), and downstream (D) exons in the pI-11 plasmid backbone. The GOLGA2 splicing reporter containing the wild-type py tract sequence was constructed by inserting a gene block containing the genomic region of *GOLGA2* (NM_001366244.2) coding for 120 bp of intron 7, exon 8 and 120 bp of intron 8. The GOLGA2 splicing reporter containing the mutated py tract sequence was constructed similarly, except the py tract of *GOLGA2* intron 7 was replaced with the py tract of *CELF1* intron 1 (NM_001330272.2) py tract. FOXP3 intron 11 reporter plasmid was constructed as previously described (16) *FOXP3* (NC_000023) intron 11 sequence was inserted into the open reading frame of *Renilla Luciferase* (Rluc) in the pcDNA3.1 vector (pcDNA3.1-Rluc).

Reporter transfections were carried out 48 hours after siRNA-mediated knockdown of DDX39A and DDX39B expression. The cells were transfected with 50 ng of IL7R and GOLGA2 splicing reporters and 500 ng of FOXP3 splicing reporter using Lipofectamine 3000 (Thermo Fisher Scientific) using the manufacturer's recommendations. Total RNA was isolated 24 hours after transfection using Direct-zol RNA kit (Zymo Research).

### Cellular fractionation

Hela cells were washed once in PBS and then scraped in PBS. The cell suspension was centrifuged at 200g for 5 min. The resulting pellet was resuspended in buffer (20 mM Tris–HCl pH7.5, 150 mM NaCl, 1 mM DTT and 1× protease inhibitor). Equal volumes of 0.15% NP-40 in PBS was added to the cell suspension and incubated for 2.5 min on ice. Cell lysates were then layered on top of 2.5 volumes of sucrose cushion [24% (w/w) sucrose, 20 mM Tris–HCl pH7.5, 150 mM NaCl, 1 mM DTT and 1× protease inhibitor] and centrifuged at 14 000 rpm for 10 min. The supernatant was collected as the cytoplasmic fraction for protein and RNA extraction. Pelleted nuclei were washed twice with PBS Centrifuge at 14 000 rpm for 5 min. After two PBS washes, the pelleted nuclei were treated with glycerol nucleoplasm lysis buffer [50% (v/v) glycerol, 20 mM Tris–HCl pH7.5, 75 mM NaCl, 0.5 mM EDTA, 1 mM DTT and 1× protease inhibitor] and mixed gently. The urea nuclei lysis buffer [1% (v/v) NP-40, 1 M urea, 20 mM HEPES pH7.5, 1 mM DTT, 7.5 mM MgCl_2_, 0.2 mM EDTA and 1× protease inhibitor] was then added to the suspension and incubated for 2 min on ice. The nucleoplasm fraction was separated by centrifugation at 14 000 rpm for 2 min. Supernatants were collected as nucleoplasm fraction and the pellets were collected as the chromatin fraction for protein and RNA extraction. Successful separation of subcellular fractions was confirmed by western blot with cytoplasmic α-Tubulin (Cell Signaling Technology, AB_1904178) and nucleoplasmic Nucleolin (Abcam, ab134164). RNA was isolated from each fraction using the Direct-zol RNA kit (Zymo Research), and 200 ng of RNA from each compartment was used for reverse transcription with random primers as above. The abundance of DDX39A intron 6, DDX39B intron 6 and GAPDH transcripts were quantified in each fraction by RT-qPCR and normalized to total RNA as above. The percentage of GAPDH, DDX39A intron 6, and DDX39B intron 6 RNA in each compartment was calculated by dividing by the corresponding total signal from the three compartments.

### Western blot analyses

Total protein was harvested using 1X RIPA buffer (150 mM NaCl, 1% NP-40, 0.5% sodium deoxycholate, 0.1% SDS and 50 mM Tris–HCl at pH 7.5) freshly supplemented with 1× protease inhibitors (Roche). 15 μg of total protein were loaded per lane on NuPAGE 4%–12% Bis–Tris pre-cast gels (Thermo Fisher Scientific), transferred to nitrocellulose membranes (Whatman), and blotted using standard protocols with anti-DDX39B rabbit polyclonal antibody (Abcam, ab47955), anti-DDX39A (Proteintech, 11723-1-AP) and anti-actin mouse monoclonal antibody (Santacruz, sc-47778) as a loading control. All western blots were quantified in Image J using densitometric analysis, and protein band intensities were normalized to actin intensities.

### RT-PCR and RT-qPCR analysis

Total RNA was isolated from control or knockdown cells using Direct-zol RNA kit (Zymo Research) and treated in-column with DNase I following the manufacturer's recommendations. Reverse transcription was conducted with 1μg of total RNA and random primers using the High Capacity cDNA Reverse Transcription Kit (Thermo Fisher Scientific). 20 μl of the reaction mix (10 μl of mastermix -prepared as per manufacturer's protocol and 10μl of diluted RNA) was used for each cDNA synthesis. The cDNA synthesis conditions were as follows: 25°C for 10 min; 37°C for 120 min; 85°C for 5 min and hold at 4°C. The Minimum Information for Publication of Quantitative Real-Time PCR Experiments (MIQE) guidelines were followed for RT-qPCR analysis (27). All qPCR reactions were set up using the SYBR Select Master Mix (Thermo Fisher Scientific), 50 ng of cDNA and 0.2 μM each of forward and reverse primer. The qPCR reactions were run on standard thermal cycling conditions on the StepOnePlus Real-Time PCR system (Thermo Fisher Scientific). The thermal cycling conditions were 50°C for 2 min (UDG activation), 95°C for 2 min (AmpliTaq DNA Polymerase activation), and 40 cycles of 95°C for 15 s (Denaturation) and 60°C for 1 min (Anneal/Extension). All primer sequences used for RT-qPCR analysis are tabulated in Supplementary File 5. GAPDH, EEF1A and β-ACTIN were used as reference genes for normalizing transcript levels in whole cell lysates since their expression was not affected by DDX39A and DDX39B depletion. MALAT1 was used as a reference gene for normalizing control for transcript levels in the nuclear fraction. 18S rRNA was used as a reference gene as a normalizing control for transcript levels in the cytoplasmic fraction.

To quantify the splicing efficiency of all the minigene reporters, endpoint PCR was carried out using the 2× Taq Master Mix (New England Biolabs) and 0.2 μM each of forward and reverse primers specific for each reporter. T7 forward primer and SP6 reverse primer were used for measuring the splicing efficiency of the *IL7R* exon 6 and *GOLGA2* exon 8 minigene reporters in the pI-11 vector backbone. The PCR reactions were run on the Biorad C1000 thermal Cycler and the thermal cycling conditions were 95°C for 30 s, followed by 20 cycles of 95°C for 30 s, 53°C for 30 s, 68°C for 30 s and final extension at 68°C for 5 min. *FOXP3*-intron 11 forward and reverse primers were used for measuring the splicing efficiency of *FOXP3* intron 11 reporters at thermocycling conditions:95°C for 30 s, followed by 18 cycles of 95°C for 30 s, 59°C for 30 s, 68°C for 30 s and final extension at 68°C for 5 min. Endogenous levels of *IL7R* and *GOLGA2* isoforms were measured using the respective primers at thermocycling conditions 95°C for 30 s, followed by 18 cycles of 95°C for 30 s, 55°C for 30 s, 68°C for 30 s and final extension at 68°C for 5 min. All primer sequences used for endpoint PCR analysis are listed in Supplementary File 5. PCR products were detected by electrophoresis on 6% TBE gel and stained with 1× SYBR Gold Nucleic Acid Gel Stain (Thermo Fisher Scientific) for 10 min. The splicing efficiency of each condition was measured by densitometry analysis in ImageJ and quantified as (spliced/(spliced + unspliced)) × 100 and then normalized to NSC.

### Statistical analyses

In all figures, error bars represent standard deviation (S.D.) unless otherwise noted. Asterisks denote level of statistical significance: ****P ≤ 0.0001; ***P ≤ 0.001; **P ≤ 0.01; *P ≤ 0.05; ns = not significant. Statistical analyses were conducted in Prism8 (GraphPad Software). Student's *t* test was used for calculating the statistical significance of the peptide level differences between DDX39A knockdown (siD06 and siD09), DDX39B knockdown (siD11 and siD13) with respective controls (NSC-5nM and NSC-25nM). Student's *t* test was used for calculating the statistical significance of the transcript level differences for comparing DDX39A knockdown (siD09), DDX39B knockdown (siD13) with control (NSC) conditions. Kruskal-Wallis test was used for statistical analyses of sequence comparisons between knockdown and control conditions. For RT-qPCR analysis in rescue experiments, a one-way ANOVA model was used, followed by the Tukey-Kramer test. For RT-qPCR analysis of DDX39A and DDX39B intron 6-retained transcript levels in THP1 cells student's t test was used.

## Results

### DDX39A and DDX39B are uniquely required for the expression of a limited number of genes.

Previous work on *IL7R* exon 6 (15) and *FOXP3* introns (16) suggested the existence of splicing events that require DDX39B, but not DDX39A, and this led us to investigate the overlap between these two paralogs. Other groups have explored the roles of DDX39A and DDX39B on RNA export and splicing. Studies have shown that siRNA-mediated depletion of DDX39A or DDX39B results in nuclear retention of mRNAs (5,28). One study suggests that DDX39A and DDX39B regulate the export of different groups of mRNAs (5). Another study found that depletion of DDX39A and DDX39B leads to an increase in the accumulation of short and long circular RNAs, respectively, in the nucleus (4). While these studies provide some evidence for both redundancy and differences in the functions of DDX39A and DDX39B, limitations of these studies convinced us to systematically explore which transcripts were altered when either DDX39B or DDX39A are depleted.

Here, we describe salient points about the methodologies used since these helped overcome limitations of previous studies, including our own. First, we depleted DDX39A or DDX39B in the same cell line, HeLa cells, and analyzed how these perturbations altered the transcriptome using massively parallel RNA sequencing (RNAseq). It should be noted that we attempted to evaluate the impact of simultaneous knockdown of DDX39A and DDX39B, but dual knockdowns resulted in marked loss of cell viability, rendering the data unreliable.

Second, in order to assuage concerns about off-target effects due to idiosyncratic behavior of siRNAs we employed two independent siRNAs to silence each protein: siD06 and siD09 targeted DDX39A and siD11 and siD13 targeted DDX39B. When analyzing our RNAseq data we considered all DDX39A depleted conditions (siD06 and siD09- treated cells) as replicates and all DDX39B depleted conditions (siD11 and siD13-treated cells) as replicates. This strategy, which we have used previously (29), reduces the number of false positive targets identified (see Materials and Methods).

Third, since the efficiency of RNAi-mediated knockdowns varies dramatically depending on the gene product being depleted and the siRNA used, we worked out conditions that led to similar level of knockdown for DDX39A and DDX39B (see Materials and Methods). To deplete DDX39A we utilized 25 nM of siD06 or siD09 and these conditions were compared to cells treated with 25 nM of the non-silencing control (NSC) siRNA (labeled NSC-25nM). To reduce DDX39B levels equivalently we used 5 nM of siD11 or siD13 and these conditions were compared to cells treated with 5 nM of the NSC siRNA (labeled NSC-5nM).

Fourth, given the questionable specificity of anti-DDX39A/B antibodies, we utilized targeted mass spectrometry to accurately determine the expression of DDX39A and DDX39B proteins after siRNA-mediated depletion. Quantification of a tryptic peptide that differs by one amino acid in DDX39A (HFVLDECDK) or DDX39B (HFILDECDK) was used to ascertain degree of DDX39A or DDX39B knockdown (Supplementary Figure S2A). We also confirmed that DDX39A or DDX39B expression was reduced at the transcript level in samples treated with siRNAs targeting DDX39A or DDX39B, respectively (Supplementary Figure S2B). These results confirmed the specific knockdown of DDX39A or DDX39B with the siRNAs that targeted DDX39A or DDX39B mRNAs, respectively (Supplementary Figure S2A & B).

To identify transcripts altered in overall expression upon depletion of DDX39A or DDX39B we analyzed the RNAseq data using DESeq2 (24). Principal component analysis revealed that all individual conditions clustered tightly (Supplementary Figure S2C). We noted that the NSC-5nM and NSC-25nM conditions also clustered close together suggesting that the two control conditions had very similar effects on the transcriptome. Additionally, the pair of siRNAs used to knockdown DDX39A or DDX39B, respectively, did not cluster together, which is likely due to well-known idiosyncratic off-target effects of siRNAs (Supplementary Figure S2C). This highlights the importance of our stringent approach that identified targets commonly altered by both siRNAs targeting each paralog.

**Figure 1.**
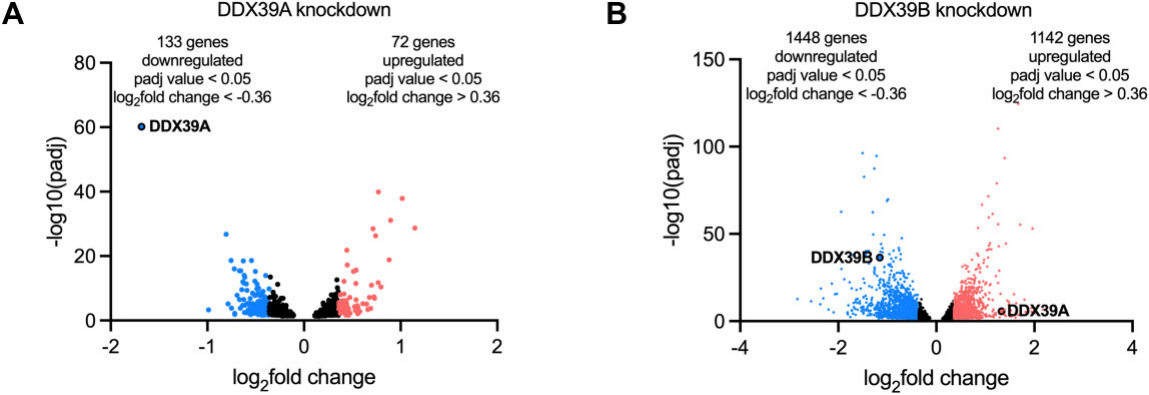
Genes differentially regulated upon DDX39A and DDX39B depletion: Volcano plots of genes differentially expressed upon **(A)** DDX39A knockdown or **(B)** DDX39B knockdown. Upregulated genes (*P*_adj_ value < 0.05 and log_2_fold change > 0.36) are represented in red, and downregulated genes (*P*_adj_ value < 0.05 and log_2_fold change <−0.36) are represented in blue.

We observed that the depletion of DDX39A resulted in the upregulation of 72 and the downregulation of 133 transcripts relative to levels observed in NSC-25nM control cells (|fold change| > 1.3; *P* < 0.05; Figure 1A & Supplementary File 1). Depletion of DDX39B led to the upregulation of 1142 and the downregulation of 1448 transcripts (|fold change| > 1.3; *P* < 0.05; Figure 1B & Supplementary File 2). The number of transcripts that showed altered expression upon DDX39B depletion was higher than those altered upon DDX39A depletion, even though the extent of knockdown was similar for the two ATPases (Supplementary Figure S2A). Additionally, depletion of DDX39B resulted in a greater relative fold-change in the expression of its target transcripts. Importantly, knockdown of DDX39A or DDX39B resulted in a modest number of changes in gene expression, which is surprising given the essential roles these proteins play in RNA metabolism. This paradox, however, could be explained by a high level of redundancy.

### DDX39A and DDX39B have overlapping roles in controlling gene expression.

**Figure 2.**
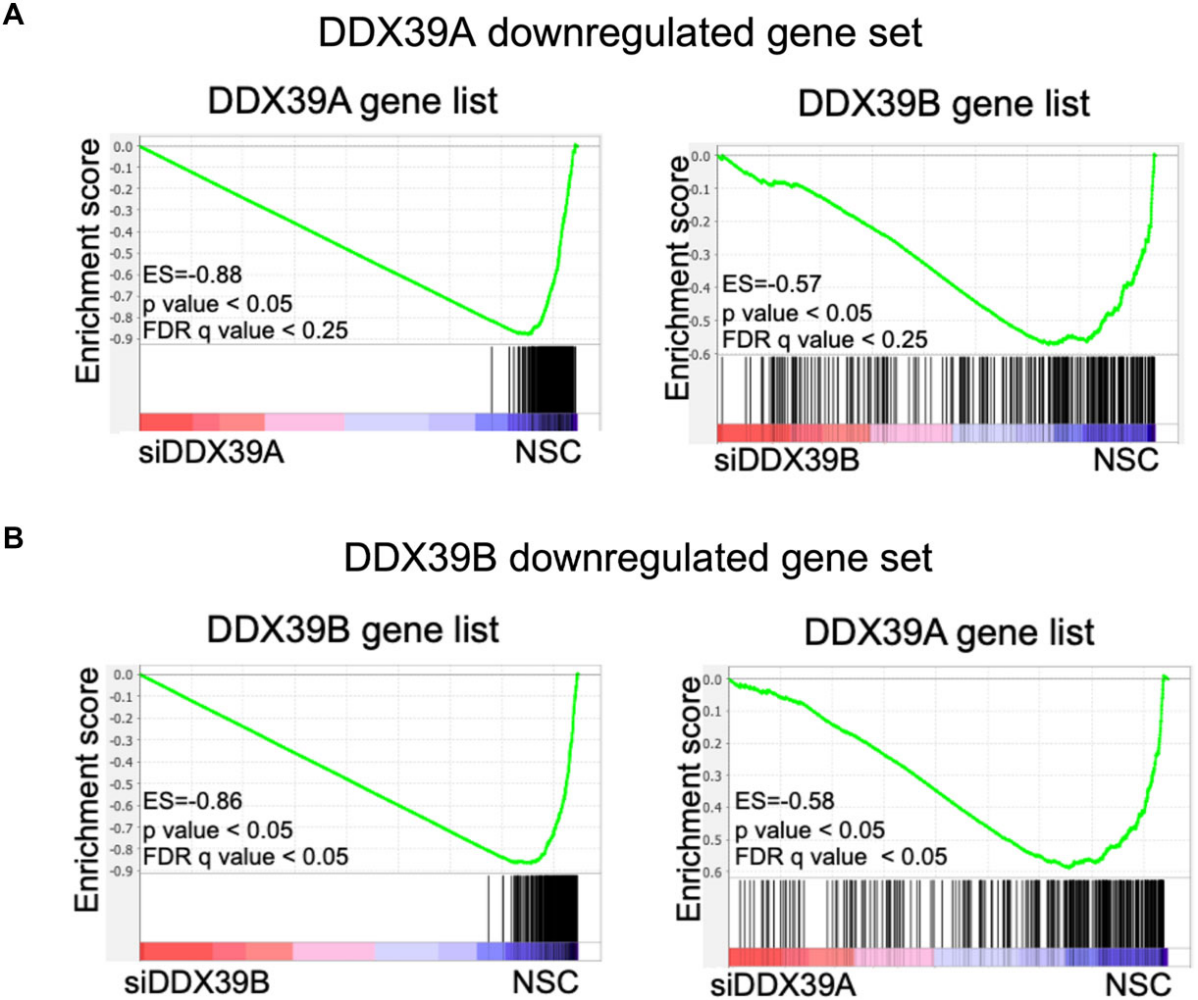
Overlap between genes regulated by DDX39A or DDX39B expression: (**A**) GSEA results of enrichment of DDX39A downregulated genes in control (NSC) over DDX39A-knockdown (left panel) and DDX39B-knockdown (right panel) in HeLa cells. (**B**) GSEA results of enrichment of DDX39B downregulated genes in control (NSC) over DDX39B-knockdown (left panel) and DDX39A-knockdown (right panel) in HeLa cells.

To examine the extent of overlap between the transcripts that are significantly altered upon DDX39A or DDX39B depletion, we carried out the Gene Set Enrichment Analysis ((GSEA) (26)). GSEA is an analytical tool that determines the distribution of a specific gene set in a transcriptome dataset where genes are ranked based on their expression. We created two independent ranked gene lists that included all transcripts that had a significant (*P*< 0.05) fold change between controls and DDX39A or DDX39B-depleted conditions (Supplementary Files 1 and 2). We also created custom gene sets that included the upregulated or downregulated genes (*P*< 0.05, |fold change| > 1.3) upon DDX39A or DDX39B knockdown. To make sure our analysis was internally consistent we looked at the enrichment of the DDX39A downregulated gene set in the DDX39A gene list. GSEA calculates an enrichment score (ES) that quantifies the extent to which genes within a gene set are overrepresented at either the top or bottom of a gene list. A positive ES implies that genes in the gene set are ranked with the high values in the gene list, while a negative ES indicates they are with low values in the gene list. As expected, the genes in the DDX39A downregulated gene set ranked among the lowest values in the DDX39A gene list (ES = −0.88, *P* < 0.05; Figure 2A, left panel). This control demonstrates the robustness of the analysis. Interestingly, most of the genes in the DDX39A downregulated gene set also had low rank values in the DDX39B gene list (ES = −0.57; *P* < 0.05; Figure 2A, right panel). The asymmetric distribution of DDX39A downregulated transcripts on the DDX39B gene list strongly suggests that a large proportion of these transcripts are also downregulated upon DDX39B knockdown. Equally, we found the 72 most upregulated transcripts upon DDX39A knockdown enriched among upregulated transcripts in the DDX39B gene list (Supplementary Figure S3A).

We evaluated the mapping of the 500 most downregulated transcripts upon DDX39B knockdown on the DDX39A and DDX39B gene lists. We used 500 transcripts instead of the 1448 identified above (Figure 1B & Supplementary File 2) because GSEA gene sets are limited to 500 genes. As previously explained, we also ran an analysis to test for internal consistency evaluating the enrichment of the 500 most downregulated transcripts upon DDX39B knockdown in the DDX39B gene list. As expected, the genes from the DDX39B downregulated gene set had the lowest ranks in the DDX39B gene list (ES = −0.86; *P* < 0.05; Figure 2B, left panel). Most important is to note that the 500 downregulated DDX39B transcripts mapped towards the downregulated genes on the DDX39A gene list (ES = −0.58, *P* < 0.05; Figure 2B, right panel). Additionally, we found the 500 most upregulated transcripts upon DDX39B knockdown enriched among upregulated transcripts in the DDX39A gene list (Supplementary Figure S3B). Again, the asymmetric distribution of the most altered transcripts upon DDX39B kd on the DDX39A gene list strongly suggests that a large proportion of genes regulated by DDX39B knockdown are also regulated by DDX39A knockdown. Overall, these observations strongly suggest that DDX39A and DDX39B have largely overlapping roles in regulating global transcript level changes.

### DDX39B and DDX39A regulate diverse alternative splicing events

**Figure 3.**
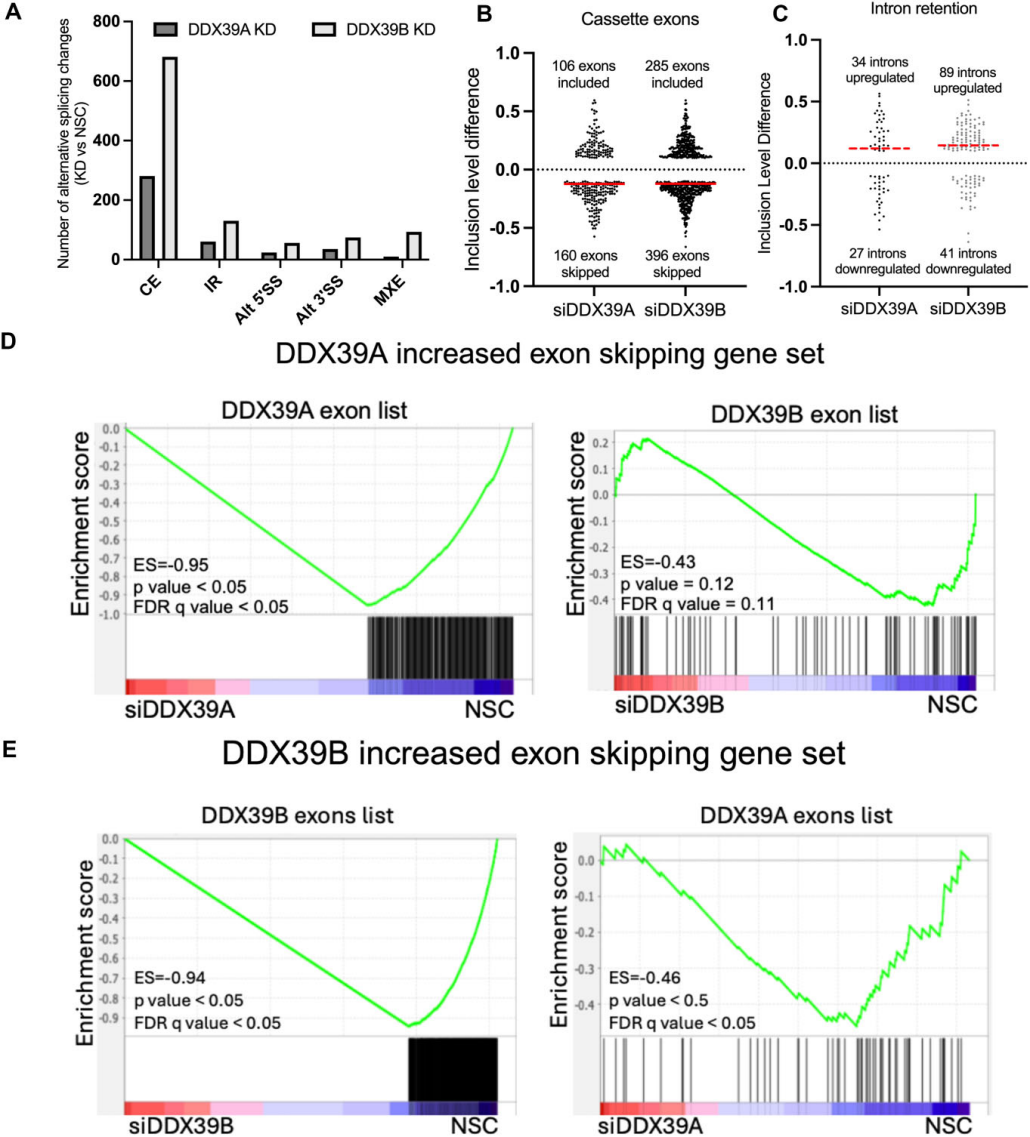
Changes in RNA alternative splicing upon DDX39A and DDX39B depletion: (**A**) number of alternative splicing events that are significantly different (*P*< 0.05 and |inclusion level difference| > 0.1) between DDX39A or DDX39B knockdown as compared to control (NSC) (CE: cassette exon splicing; IR: intron retention; Alt3’SS: alternative 3′splice site; Alt5’SS: alternative 5′SS; MXE: mutually exclusive exons). (**B**) Inclusion level differences of exons that are differentially spliced upon DDX39A and DDX39B knockdown. A positive inclusion level difference indicates exons that are included more upon knockdown, and a negative inclusion level difference indicates exons that are skipped more (included less) upon knockdown. (**C**) Inclusion level differences of introns differentially spliced upon DDX39A and DDX39B knockdown. A positive inclusion level difference indicates introns that are retained (included more) upon knockdown, and a negative inclusion level difference indicates introns that are spliced (included less) more upon knockdown. The red line indicates the median inclusion level difference. (**D**) GSEA results of enrichment of exons skipped more upon DDX39A knockdown in NSC control compared to DDX39A knockdown (left panel) and DDX39B knockdown (right panel). (**E**) GSEA results for enrichment of exons skipped more upon DDX39B knockdown in controls compared to DDX39B knockdown (left panel) and DDX39A knockdown (right panel).

Given what DDX39A and DDX39B are involved in constitutive and alternative RNA splicing, we used rMATS (25) analysis of our RNAseq data to determine global changes in RNA splicing caused by DDX39A or DDX39B depletion. As done above for transcript level changes, we only considered splicing changes common to the two siRNAs used for silencing DDX39A or DDX39B respectively. Altered splicing events were defined as those with |inclusion level difference| ≥ 0.1 (10% change), *P* < 0.05 and FDR < 0.01, when comparing DDX39A or DDX39B knockdowns relative to NSC controls. All categories of alternative RNA splicing, including cassette exon use (CE), intron retention (IR), alternative 3′ and 5′ splice site usage (Alt 5′SS and Alt 3′SS), and mutually exclusive exon (MXE) splicing, were impacted when DDX39A or DDX39B were depleted (Figure 3A). A complete list of all the alternative splicing events affected by DDX39A or DDX39B depletion are provided in Supplementary Files 3 and 4, respectively. Several general observations are noted. We observed a higher number of RNA splicing changes upon knockdown of DDX39B compared to knockdown of DDX39A. The most common events changed upon depletion of either ATPase are cassette exon use (CE) and intron retention (IR) in that order (Figure 3A). Upon DDX39A or DDX39B knockdown more cassette exons are less efficiently included than *vice versa* (Figure 3B). For instance, in cells depleted of DDX39B, 396 cassette exons have reduced inclusion level versus 285 with increased inclusion level (Figure 3B). When we examine the direction of the effect among intron retention events, we note that upon DDX39A or DDX39B knockdown more introns are less efficiently removed (thus higher intron inclusion levels) than *vice versa* (Figure 3C). The direction towards less efficient splicing, which for cassette exons is less inclusion and for retained introns is more inclusion, is consistent with the known activity of DDX39B and the presumed activity of DDX39A. As observed for transcript level changes we note that the fraction of splicing events that significantly change with DDX39A or DDX39B depletion is relatively small, and this could be explained by significant redundancy between DDX39A and DDX39B.

To address the possibility of redundancy we used GSEA as we have done above, except we compared sets of splicing events (equivalent to GSEA gene sets) to lists of splicing events ranked by magnitude of the splicing change (equivalent to GSEA gene lists) (See Methods). Our analysis suggests that there is some overlap among cassette exons that are dependent on DDX39A and DDX39B (Figure 3D and E), but the data are not as robust as the overlap observed for transcript level changes (Figure 2). The data also suggest that a small subset of exons may be regulated in opposite directions by the two ATPases (Figure 3D), but this was not explored further. We could not find evidence for overlap among intron retention events (Supplementary Figure S4).

### The splicing of *IL7R* exon 6 is controlled by DDX39B, not DDX39A.

**Figure 4.**
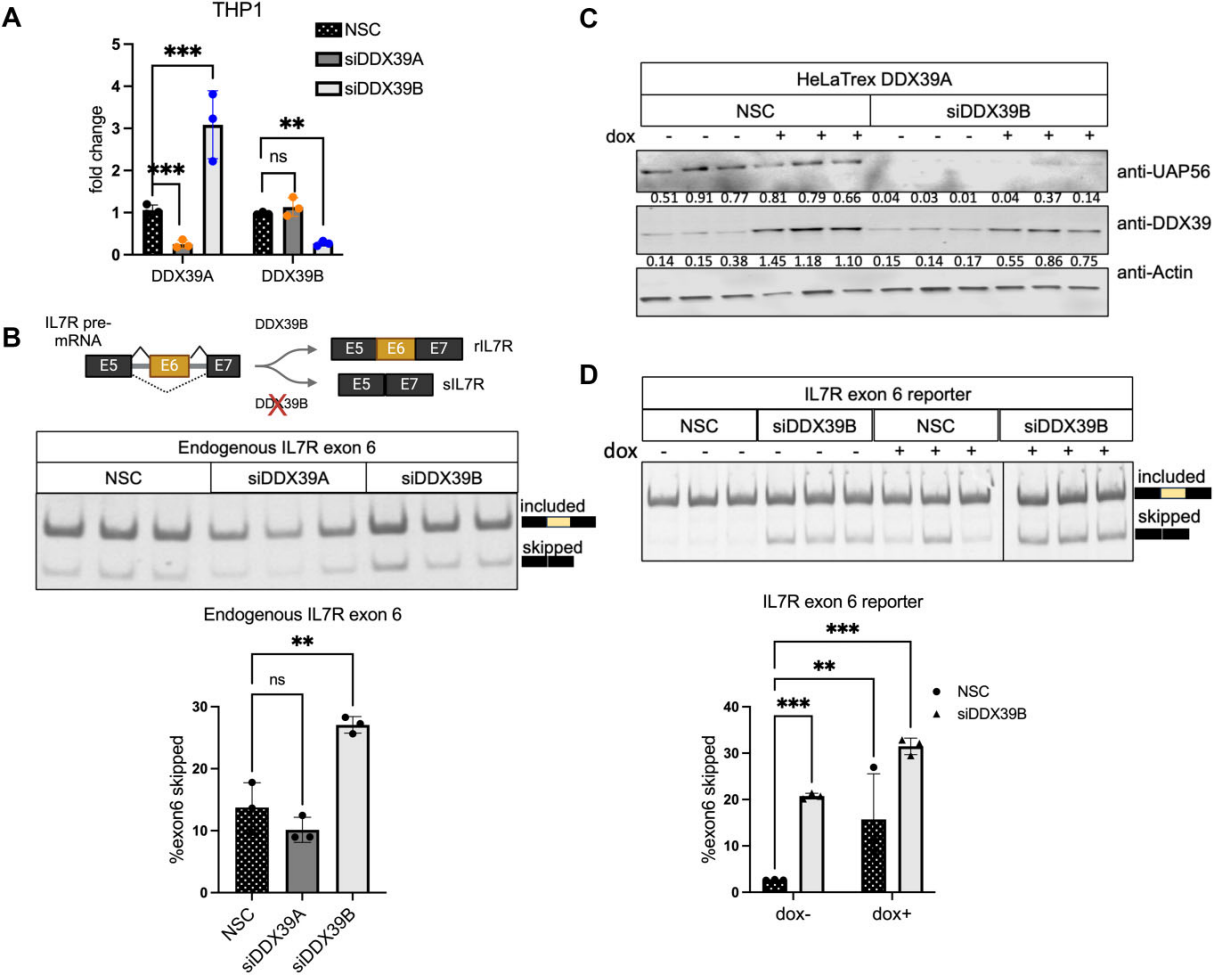
DDX39A does not regulate *IL7R* exon 6 splicing. (**A**) RT-qPCR analysis of the transcript levels of DDX39A and DDX39B upon DDX39A knockdown (siD09) and DDX39B knockdown (siD13) compared to control (NSC) in THP1 cells. Statistical significance was assessed using Student's *t* test (***P ≤ 0.001; **P ≤ 0.01; ns = not significant). (**B**) The top panel provides a diagrammatic representation of *IL7R* exon 6 splicing in the presence and absence of DDX39B. The bottom panel shows the RT-PCR analysis of *IL7R* exon 6 splicing in transcripts from endogenous *IL7R*. (**C**, **D**) Rescue experiments of IL7R exon 6 splicing in HeLa cells stably expressing siRNA-resistant DDX39A transgene under the control of doxycycline promoter. (C) Immunoblot depicting the protein abundance of DDX39A and DDX39B with respect to actin. The numbers indicating protein abundance relative to actin are listed below each immunoblot. (D) RT-PCR analysis of IL7R exon 6 splicing from minigene reporter. In all panels, the data are shown as mean ± s.d., and statistical significance was assessed using one-way ANOVA (****P ≤ ***P ≤ 0.001; **P ≤ 0.01; ns = not significant).

The data above suggested some degree of overlap for cassette exons regulation by the two ATPases, but also a significant number of cassette exons uniquely dependent on DDX39B for efficient inclusion. We have previously shown that DDX39B is an activator of *IL7R* exon 6 splicing and that depleting DDX39B promotes the skipping of exon 6 in both HeLa cells and primary human CD4^+^ T cells (15). The exquisite sensitivity of *IL7R* exon 6 inclusion to DDX39B levels suggested a requirement that DDX39A could not complement. To directly test whether or not DDX39A could impact *IL7R* exon 6 splicing we carried out siRNA-mediated depletion of DDX39A or DDX39B in THP1 cells, a monocytic cell line that expresses both IL7R isoforms. The levels of DDX39A and DDX39B transcripts decreased significantly following knockdown with siDDX39A (siD09) and siDDX39B (siD13), respectively (Figure 4A). As observed in the RNAseq data (Figure 1), there was a greater than two-fold increase in DDX39A transcript levels when DDX39B was depleted, suggesting that DDX39B represses DDX39A expression, but not *vice versa* (Figure 4A). Other studies that carried out siRNA-mediated knockdowns of DDX39A and DDX39B also reported an increase in DDX39A transcript levels when DDX39B is depleted (5,6,28). At the protein level, DDX39B expression was reduced by more than 50% in cells transfected with siDDX39B, compared to cells transfected with the non-silencing control (NSC) or siDDX39A (Supplementary Figure S5A).

We measured the expression of endogenous *IL7R* transcript isoforms that include or skip exon 6 using RT-PCR analysis. We observed that depleting DDX39B resulted in a two-fold increase in *IL7R* exon 6 skipping when compared to NSC-treated cells (Figure 4B), which is consistent with our previous observations (15). In contrast, depleting DDX39A did not increase the skipping of *IL7R* exon 6 (Figure 4B). We further confirmed these observations in HeLa cells using an *IL7R* exon 6 minigene reporter system (15,18). We observed a similar increase in *IL7R* exon 6 skipped transcripts from the minigene reporter following DDX39B depletion but not with DDX39A depletion (Supplementary Figure S5B).

Since DDX39B is also involved in the nuclear export of mRNAs, we wanted to determine whether the disruption of nuclear export of these mRNAs upon DDX39B depletion mediates the differences in *IL7R* transcript isoform levels. We measured the relative abundance of these transcript isoforms in different subcellular fractions (Supplementary Figure S5C) upon DDX39A or DDX39B depletion. We observed a significant increase in the levels of the exon6-skipped *IL7R* transcript isoform and a corresponding decrease in the *IL7R* exon6-included isoform in both the nuclear and cytoplasmic fractions. These observations suggest that the changes in *IL7R* transcript isoform levels are due to a defect in DDX39B-mediated RNA splicing, rather than nuclear export (Supplementary Figure S5D).

We previously showed that overexpressing a siRNA-resistant DDX39B cDNA transgene in DDX39B-depleted conditions rescues *IL7R* exon 6 splicing (15). We investigated whether DDX39A overexpression could rescue *IL7R* exon 6 splicing in the absence of DDX39B. We generated a HeLaTrex cell line that expresses a siRNA resistant DDX39A cDNA transgene under doxycycline control. Upon doxycycline stimulation, we observed an increase in DDX39A expression in both control and DDX39B-depleted conditions (Figure 4C). Depletion of DDX39B in the DDX39A-overexpressing cell line led to a significant increase in *IL7R* exon 6 skipping in the absence of doxycycline (Figure 4D). Overexpression of DDX39A failed to rescue *IL7R* exon 6 splicing in DDX39B-depleted condition. In fact, it led to a greater increase in *IL7R* exon 6 skipping (Figure 4D). Interestingly, overexpressing DDX39A in control conditions also increased *IL7R* exon 6 skipping by approximately 3-fold, suggesting that overexpression of DDX39A has a dominant negative effect on *IL7R* exon 6 splicing (Figure 4D). Overexpression of DDX39A could inhibit interactions of DDX39B by sequestering common factors, possibly explaining the dominant negative effect on *IL7R* exon 6 splicing in the control conditions. In addition, overexpressed DDX39A could heterodimerize with DDX39B and thereby inhibit its function (7).

To investigate the regions of DDX39B responsible for its unique function in *IL7R* exon 6 splicing, we generated chimeras of the two ATPases by replacing the N- and C-terminal halves of DDX39A with the corresponding halves of DDX39B. The 10 aa linker motif connecting both the N- and C-terminal halves in DDX39A and DDX39B is the identical and thus is the same in both chimeras. We observed that the N-DDX39B-C-DDX39A chimera, which contains the N-terminal half (1–251 aa) of DDX39B and the C-terminal half of DDX39A (261–427 aa), cannot rescue *IL7R* exon 6 splicing when DDX39B is depleted (Supplementary Figure S6A). Similarly, the N-DDX39A-C-DDX39B chimera, which has the N-terminal half of DDX39A (1–250 aa) and the C-terminal half of DDX39B (262–428 aa), also fails to rescue *IL7R* exon 6 splicing (Supplementary Figure S6B). Overall, these observations suggest that *IL7R* exon 6 splicing uniquely depends on DDX39B activity, not DDX39A, and sequence motifs in both the N- and C-terminal halves of DDX39B are required for this activity.

### DDX39B-dependent cassette exons have U-poor/C-rich py tracts in their upstream introns.

**Figure 5.**
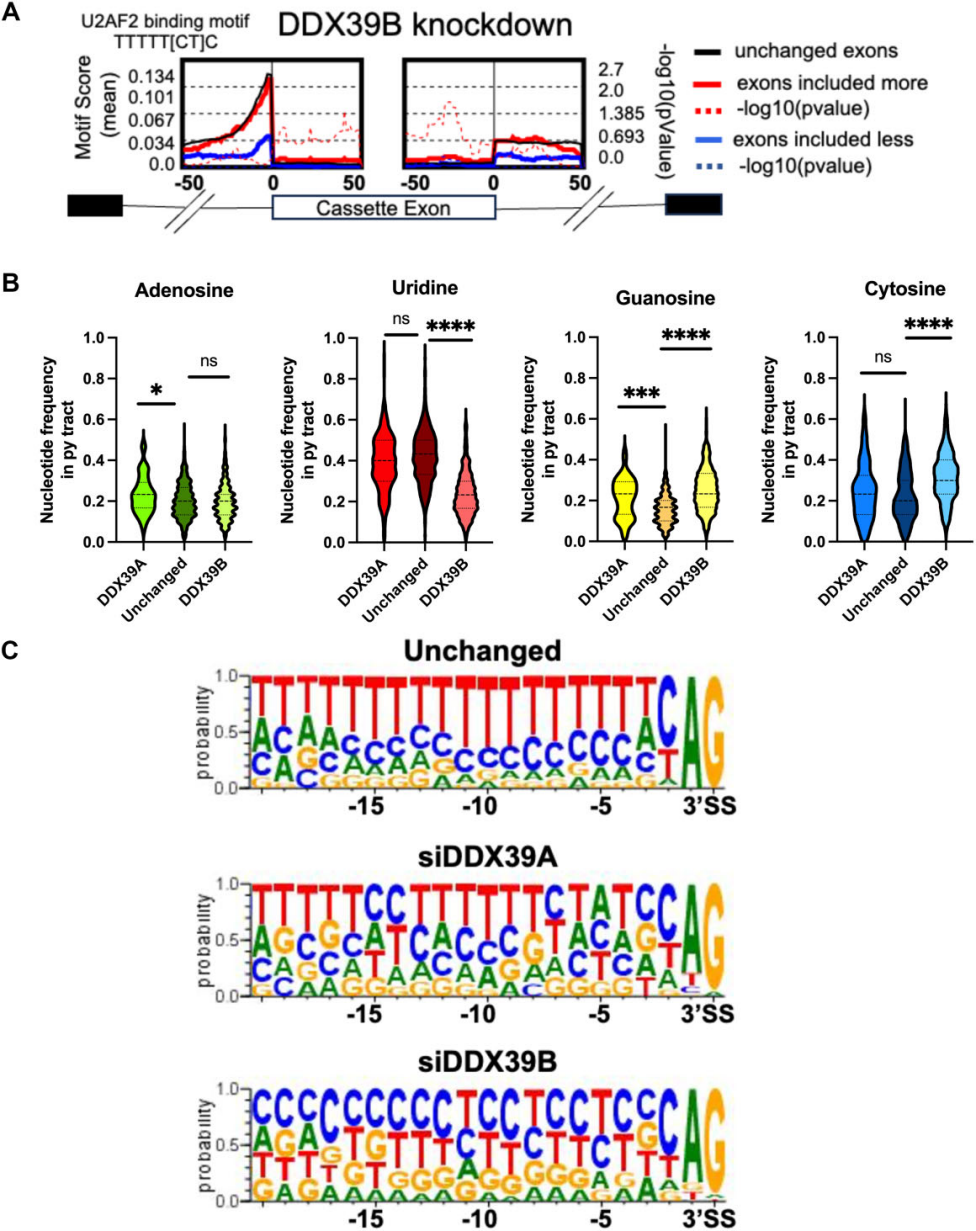
Exons sensitive to DDX39B have U-poor/C-rich py tracts in their upstream introns. (**A**) rMAPS analysis showing the enrichment of U2AF2 binding motif in introns upstream of cassette exons that are differentially regulated upon DDX39B depletion. (**B**) Violin plots depicting the nucleotide frequencies in py tracts of introns upstream of cassette exons skipped more (included less) upon DDX39A (*n* = 160) and DDX39B (*n* = 396) depletion compared to exons that are unchanged (*n* = 150) upon either knockdown. Statistical significance is calculated using Kruskal–Wallis test (****P ≤ 0.0001; ***P ≤ 0.001; *P ≤ 0.05; ns = not significant). (**C**) Sequence comparisons of py tracts of introns upstream of cassette exons skipped more upon DDX39A and DDX39B depletion.

We decided to investigate common features among cassette exons uniquely dependent on DDX39B. We previously showed that *IL7R* exon 6 inclusion is repressed by an exonic splicing silencer element, which includes a uridine-rich U2AF2-binding motif (UUUUU[C/U]C)(30). Using rMAPS (31) we investigated whether other exons that are less included upon DDX39B depletion also contain such U2AF2-binding motifs. We did not find enrichment of an U2AF2-binding motif within the exons that are included less (blue line) or those included more (red line) upon DDX39B depletion (Figure 5A). We did find a U2AF2-binding motif immediately upstream of unaffected (black line) cassette exons and those included more upon DDX39B depletion. This would be expected for exons preceded by introns with strong polypyrimidine (py) tracts. Interestingly, the enrichment score for the U2AF2-binding motif in the intronic region (∼30bp upstream of the 3′SS) was much lower for exons that were less included upon DDX39B depletion (Figure 5A). The lower U2AF2-binding motif score suggests that introns upstream of DDX39B-dependent cassette exons have weak py tracts, which is what we had concluded for the py tract upstream of *IL7R* exon 6 (30)

We examined the base composition of py tracts upstream of the 396 cassette exons less included with DDX39B depletion and confirmed that these py tracts are U-poor (Figure 5B). The frequency of Us in py tracts upstream of cassette exons unaffected by DDX39A or DDX39B knockdown is ∼40%, while that for the DDX39B-dependent exons is only 20%. These py tracts associated with exons that are less included upon DDX39B depletion also have higher frequency of Cs and Gs (Figure 5B). In contrast, the 160 cassette exons that are skipped more upon DDX39A knockdown have no decrease in U residues in the upstream py tract, and the only significant difference between these py tracts and those found upstream of unaffected exons is a slight increase in the frequency of purines (Figure 5B). These observations suggest that cassette exons that are specifically sensitive to DDX39A or DDX39B expression are associated with upstream py tracts that differ in their nucleotide composition (Figure 5C). Specifically, cassette exons that depend on DDX39B for efficient inclusion have upstream U-poor/C-rich py tracts (Figure 5C).

### A U-poor/C-rich py tract is required for DDX39B dependency of cassette exons.

**Figure 6.**
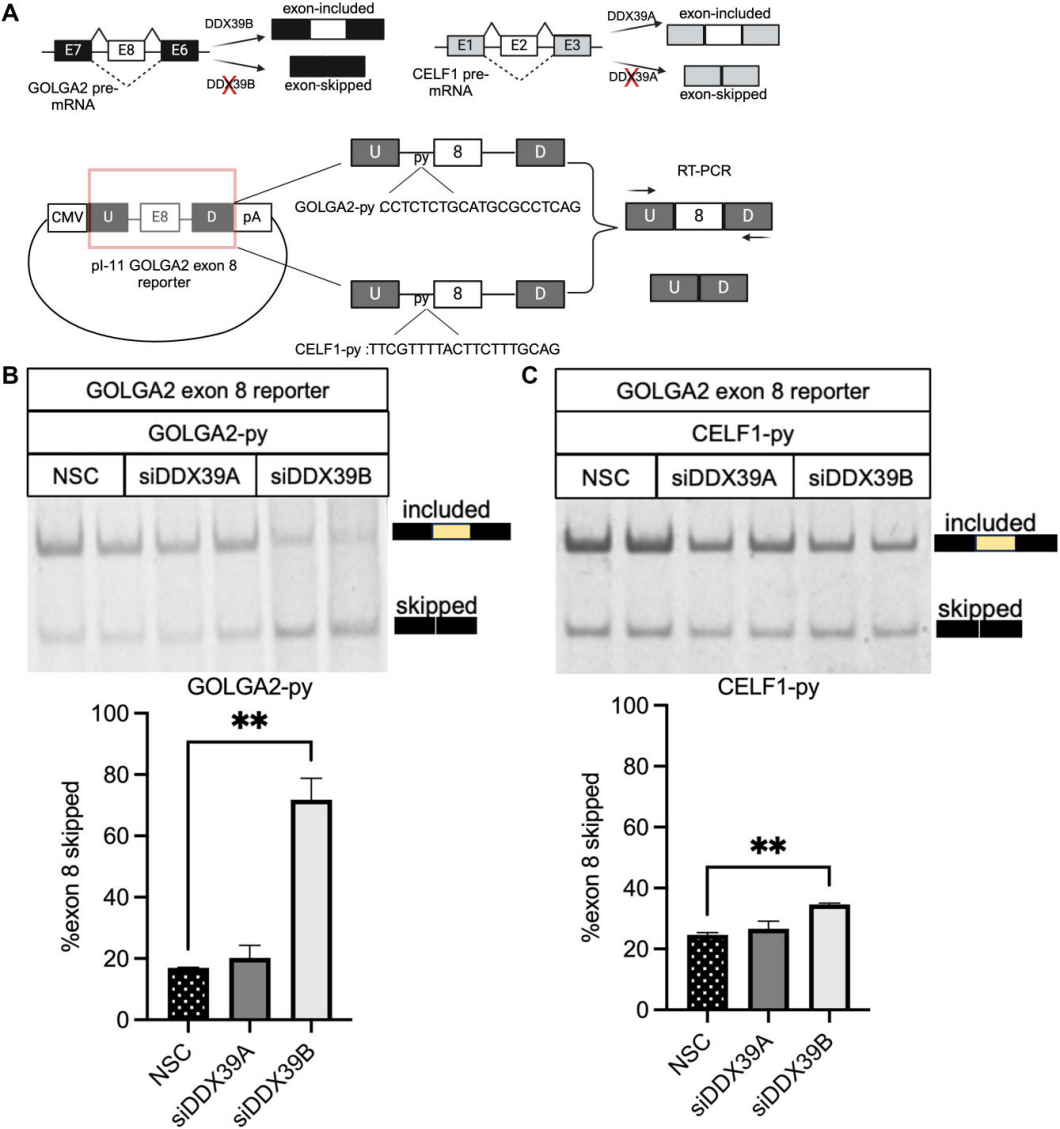
U-poor py tract of *GOLGA2* exon 8 dictates its dependency on DDX39B expression. (**A**) Top panel shows the change in alternative splicing of *GOLGA2* exon 8 and *CELF1* exon 2 upon DDX39B and DDX39A depletion, respectively. (Bottom panel) Construction of *GOLGA2* exon 8 reporters with wild-type *GOLGA2* intron 7 py tract and *CELF1* intron 1 py tract. RT-PCR analysis of GOLGA2 exon 8 reporters splicing changes with (**B**) wild-type *GOLGA2* intron 7 and (**C**) *CELF1*intron 1 py tracts in DDX39A (siD09) and DDX39B (siD13) knockdown and control cells. The top panel shows the RT-PCR amplicons on 6% TBE gels and the bottom panel shows the bar graphs depicting the percent exon 8 skipping based on quantification of these gels. Statistical significance was calculated using the Student's *t* test (**P ≤ 0.01)

We tested the functional importance of the upstream py tract on inclusion of DDX39B-dependent cassette exons. Skipping of *GOLGA2* exon 8 is increased upon DDX39B depletion, but not DDX39A depletion (Supplementary File 4 & Supplementary Figure S7A). Furthermore, *GOLGA2* exon 8 inclusion was rescued by overexpression of DDX39B but not of DDX39A (Supplementary Figure S7B). These observations suggest that splicing of *GOLGA2* exon 8 is specifically sensitive to DDX39B expression and not DDX39A. We cloned the *GOLGA2* exon 8 and the surrounding intronic sequences into the alternative splicing minigene reporter constructs pI-11 (18) (Figure 6A). RT-PCR analysis of transcripts from the minigene reporter showed that DDX39B depletion resulted in an approximately 1.5-fold increase in skipping of exon 8 compared to control or DDX39A depletion (Figure 6B). We replaced the C-rich py tract of *GOLGA2* intron 7 with the relatively U-rich py tract of *CELF1* intron 1 (Figure 6A). *CELF1* exon 2 was skipped more upon DDX39A depletion but was unaffected by DDX39B depletion (Supplementary Files 3 and 4). Replacing the py tract of *GOLGA2* intron 7 with that of *CELF1* intron 1 eliminated the sensitivity of exon 8 to DDX39B depletion, indicating that the py tract was necessary for DDX39B-dependency of *GOLGA2* exon 8 (Figure 6C). It should be noted that the *CELF1* intron 1 py tract did not make the splicing reporter sensitive to DDX39A depletion, suggesting that the py tract was not sufficient to confer DDX39A sensitivity (Figure 6C). Overall, these observations emphasize the importance of a U-poor/C-rich py tract in rendering a cassette exon sensitive to DDX39B depletion.

### Common requirement for U-poor/C-rich py tracts for DDX39B dependent cassette exons and introns.

**Figure 7.**
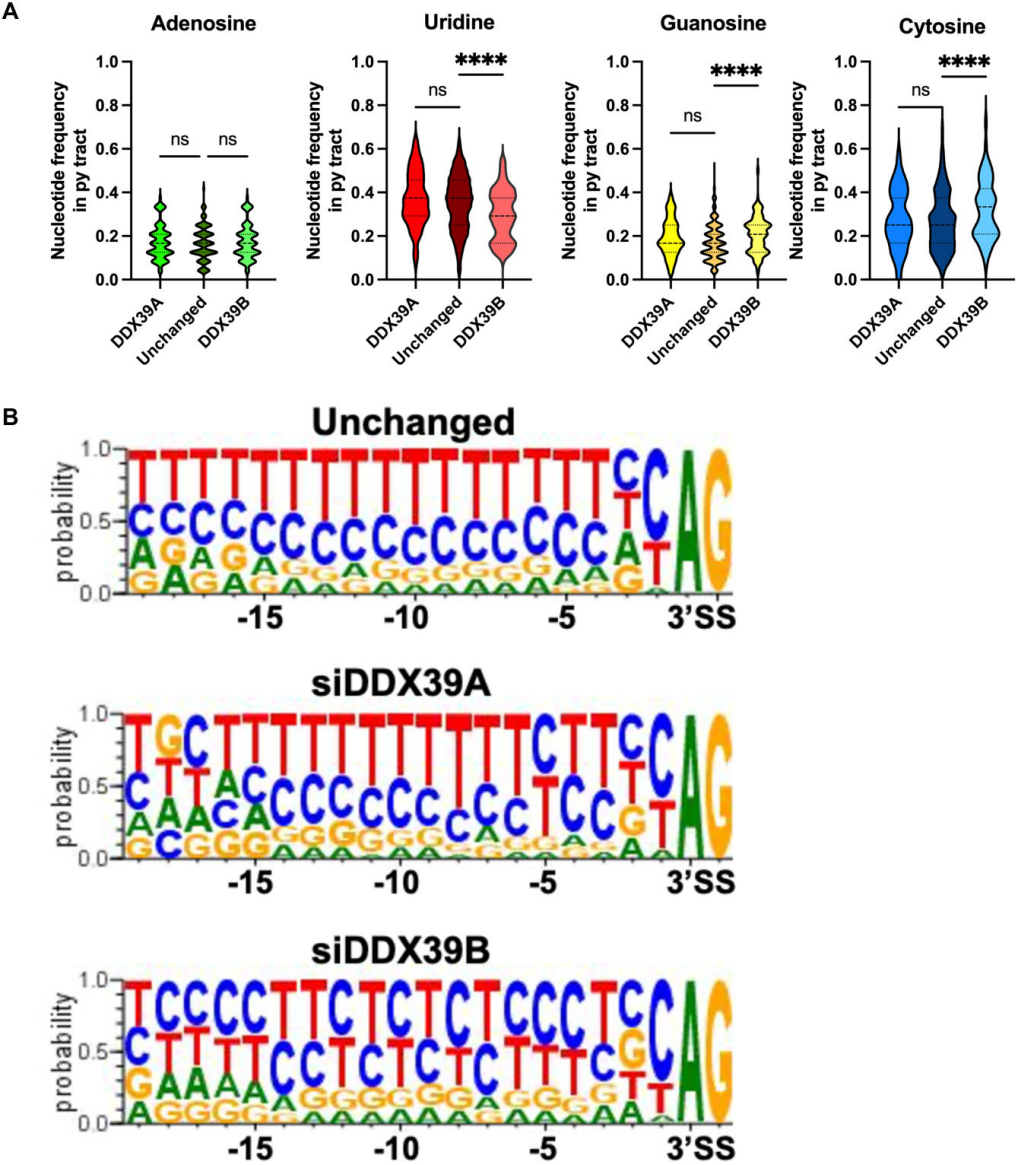
Introns dependent on DDX39B expression have U-poor/C-rich py tracts. (**A**) Violin plots depicting the nucleotide frequencies in py tracts of introns retained more upon DDX39A (*n* = 34) and DDX39B (*n* = 89) depletion compared to introns unchanged (*n* = 100) upon either knockdown. Statistical significance is calculated using Kruskal–Wallis test (****P ≤ 0.0001; ns = not significant). (**B**) Sequence comparisons of py tracts of introns retained more upon DDX39A or DDX39B depletion and unchanged introns.

The observations above for DDX39B-dependent cassette exons are similar to those we previously published for the splicing of DDX39B-dependent introns in T cells, which have U-poor/C-rich py tracts (16). To confirm this similarity in the same dataset we examined *cis*-acting elements associated with the 89 introns that were significantly more retained upon DDX39B depletion (Figure 3C). We compared these to 34 introns more retained upon DDX39A depletion (Figure 3C) and 100 introns unaltered by either knockdown. DDX39B-dependent introns, but not DDX39A-dependent ones, were U-poor and C-rich (Figure 7A). This can be visualized clearly by sequence logos of the py tracts (Figure 7B). These data recapitulate our previously published observations re DDX39B-dependent introns in T cells and lead us to conclude that U-poor/C-rich py tracts determine DDX39B dependence for both introns that contain them and cassette exons immediately downstream of them.

### DDX39A is not required for *FOXP3* intron 11 splicing, but its overexpression can rescue the requirement for DDX39B.

Our data suggested that the 89 introns that are retained more when DDX39B is depleted are not affected by DDX39A depletion, which suggested a specific need for DDX39B that could not be complemented by DDX39A. We used a *FOXP3* intron 11 splicing reporter as a model for these introns to test whether or not their splicing was sensitive to DDX39A. This reporter, which was described in Hirano *et al.*, 2023, had the *FOXP3* intron 11 inserted into the open reading frame of Renilla luciferase and quantified splicing by RT-PCR. As shown previously, when we depleted DDX39B, the splicing of *FOXP3* intron 11 significantly decreased (Supplementary Figure S8A and B). In contrast, when we depleted DDX39A, there was no significant change in the splicing efficiency of the *FOXP3* intron 11 reporter (Supplementary Figure S8A and B). We conclude from this that splicing of *FOXP3* intron 11 does not require normal levels of DDX39A.

Nonetheless, we were interested to see whether overexpressing DDX39A could rescue *FOXP3* intron 11 splicing in cells depleted of DDX39B. We depleted DDX39B and induced DDX39A overexpression by adding doxycycline (Supplementary Figure S8C). Surprisingly, overexpressing DDX39A in DDX39B depleted cells rescued the splicing of the *FOXP3* intron 11 reporter (Supplementary Figure S8D). Overall, these observations suggest that although there is no requirement for DDX39A for splicing of *FOXP3* intron 11, high levels of DDX39A can complement the requirement for DDX39B for this splicing. This is in clear contrast to the inability of DDX39A to complement inclusion of *IL7R* exon 6.

### DDX39A and DDX39B regulate the splicing of introns in their respective transcripts.

**Figure 8.**
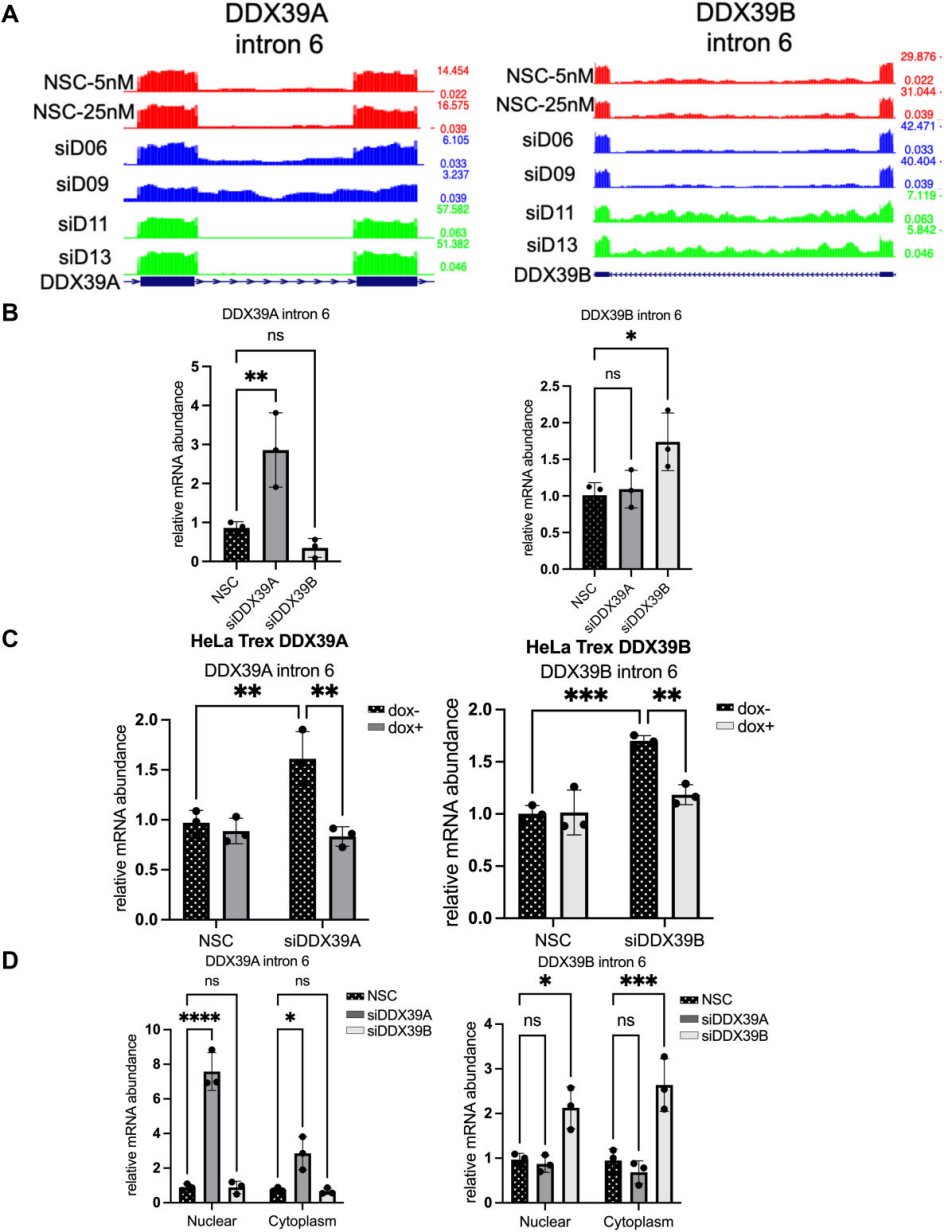
DDX39A and DDX39B regulate the splicing of their respective introns. Increase in DDX39A and DDX39B intron 6 retention upon DDX39A and DDX39B depletion, respectively. (**A**) Bed graphs depicting the read coverage for DDX39A intron 6 (left panel) and DDX39B intron 6 (right panel) in DDX39A-depleted (siD06 and siD09), DDX39B-depleted (siD11 and siD13) and control-treated (NSC-5nM and NSC-25nM) samples. (**B**) RT-qPCR analysis of the expression of DDX39A intron 6-retained transcripts relative to total DDX39A transcripts and DDX39B intron 6-retained transcripts relative to total DDX39B transcripts in DDX39A knockdown (siD09), DDX39B knockdown (siD13) and control HeLa cells. (**C**) Rescue experiments to measure the levels of DDX39A intron 6 and DDX39B intron 6 retained transcripts using RT-qPCR in HeLa cells stably expressing siRNA-resistant DDX39A or DDX39B transgene under the control of the doxycycline operator. (**D**) RT-qPCR analysis of DDX39A intron 6-retained and DDX39B-intron 6-retained transcripts in subcellular compartments. MALAT1 and 18S ribosomal RNA (rRNA) were used as normalization controls for measuring transcript levels in the nuclear and cytoplasmic fractions, respectively. In all panels, the data are shown as mean ± s.d. and statistical significance was calculated using one-way ANOVA (****P ≤ 0.0001; ***P ≤ 0.001; **P ≤ 0.01; *P ≤ 0.05; ns = not significant).

The analysis of the introns that specifically depend on DDX39A or DDX39B expression led to an unexpected observation: *DDX39A* intron 6 and *DDX39B* intron 6 are dependent on DDX39A and DDX39B, respectively (Figure 8A). We also analyzed our previous RNAseq data from DDX39B-depleted CD4^+^ T cells and found that shRNA-mediated DDX39B depletion also resulted in an increase in *DDX39B* intron 6 retention (16); data not shown). We further confirmed these observations in HeLa and THP1 cells using RT-qPCR. Depleting DDX39B resulted in a significant increase in the *DDX39B* intron 6 retained transcripts in HeLa and THP1 cells (Figure 8B and Supplementary Figure S9). Similarly, *DDX39A* intron 6 retained transcripts were higher in DDX39A-depleted HeLa and THP1 cells (Figure 8B and Supplementary Figure S9). Overexpressing DDX39A rescued *DDX39A* intron 6 splicing in DDX39A-depleted cells. Similarly, DDX39B overexpression rescued *DDX39B* intron 6 splicing in DDX39B-depleted cells (Figure 8C). Notably, the py tract of *DDX39B* intron 6 is U-poor/C-rich and has long continuous stretches of cytosines compared to *DDX39A* intron 6. These observations confirm that *DDX39A* intron 6 and *DDX39B* intron 6 specifically depend on DDX39A and DDX39B expression, respectively. This suggests a positive-feedback loop for the expression of these ATPases.

In addition to their roles in splicing, DDX39A and DDX39B are involved in the nuclear export of RNAs, and we investigated whether depleting these two ATPases impacts the nuclear export of their respective intron-retained transcripts. We performed biochemical fractionation of cellular fractions and (Supplementary Figure S5C) measured the expression of the *DDX39A* intron 6 retained and *DDX39B* intron 6 retained transcripts in these fractions. We observed a very large increase in *DDX39A* intron 6 retained transcripts in the nuclear fraction from cells depleted of DDX39A compared to either DDX39B depletion or NSC control (Figure 8D, left panel). This suggests that DDX39A depletion was affecting the splicing of *DDX39A* intron 6. Similar conclusions were reached from the equivalent data on DDX39B, depletion of this ATPase affects splicing of *DDX39B* intron 6; however, our data suggest that this transcript is transported to the cytoplasm (Figure 8D, right panel).

## Discussion

DDX39A and DDX39B are DExD-box RNA-dependent ATPases that are essential in pre-mRNA splicing and nuclear export. These two paralogs share more than 90% sequence identity and are, therefore, assumed to have similar functions. Our study demonstrated that, indeed, there is a significant redundancy in the gene targets regulated by DDX39A and DDX39B. Nevertheless, we also found that DDX39A and DDX39B differ in regulating alternative splicing of *IL7R* exon 6 and *FOXP3* introns and several other targets that are uniquely sensitive to DDX39A or DDX39B depletion. We establish that the C-richness/U-poorness of the polypyrimidine tract is one of the critical factors determining the sensitivities of introns and exons to DDX39B levels. Given the essential roles DDX39A and DDX39B have in RNA processing, it was not surprising to find an overall downregulation of gene expression when these ATPases were depleted. We establish that both DDX39A and DDX39B can, to some extent, compensate for the loss of the other paralog, which explains why the number of differentially expressed genes upon either knockdown was not high. Knocking down either DDX39A or DDX39B individually does not lead to cell death, but the simultaneous knockdown of both proteins is toxic (data not shown), further highlighting their functional redundancy. The impact of depleting DDX39A on gene expression is less severe than that of depleting DDX39B, suggesting that certain processes require DDX39B more than DDX39A. While it appears that DDX39A and DDX39B have largely redundant roles in RNA processing, it is possible that these paralogs have evolved to have tissue or cell type-specific roles. In most tissues, DDX39B expression is higher than DDX39A, but in the testis DDX39A has a higher expression than DDX39B in both mice and men (3). DDX39A expression, but not DDX39B, is also higher in proliferating lung squamous carcinoma cells (32). Interestingly, in *Xenopus laevis* DDX39A expression is higher in the subpopulation of proliferating cells in developing and regenerative tissues (33). One can speculate whether DDX39A activity has more cell cycle specific roles in proliferating cells than DDX39B. Evidence suggests that DDX39A and DDX39B regulate the nuclear export of different subsets of mitotic factors (5). Our prior work and data presented here indicate that DDX39B plays specific roles in immune modulation not shared by DDX39A.

Like cassette exons, introns with C-rich py tract depend more on DDX39B than DDX39A for splicing. Py tracts with higher frequencies of cytosines instead of uracil have weaker binding affinity for U2AF2 (34), and we proposed that C-rich py tracts depend on DDX39B ATPase activity to form stable U2AF interactions and aid in subsequent steps of spliceosome assembly (16,22). *FOXP3* introns with C-rich py tracts are specifically retained upon DDX39B depletion and not DDX39A, but an overexpression of DDX39A in the absence of DDX39B is able to rescue *FOXP3* intron splicing. It is possible that under normal conditions, DDX39B is more likely to form favorable interactions with U2AF2 on C-rich py tracts than DDX39A. Only an overabundance of DDX39A under DDX39B-depleted conditions allows for favorable interactions between DDX39A and U2AF2 due to the mass action effect. Interestingly, overexpression of DDX39A did not rescue *IL7R* exon 6 splicing under DDX39B-depleted conditions. We speculate that while an overabundance of DDX39A can compensate for the DDX39B ATPase activity, it cannot compensate for the DDX39B helicase activity, which is required for *IL7R* exon 6 splicing (15). Although DDX39A and DDX39B have structurally conserved helicase core domains, we cannot assume that they have identical ATPase and helicase activities. The reason being the enzymatic activities of these proteins may be influenced by interactions with other factors. In fact, the RNA-binding and enzymatic activities of DExD-box proteins can be regulated by their interacting partners. For example, the interaction between eIF4A and eIF4G stimulates eIF4A’s helicase activity, which is otherwise weak (35). DDX39A and DDX39B are known to interact with different proteins, CIP29 and Tho1, respectively, to form nuclear export complexes that recognize and transport specific subsets of mRNAs and circRNAs (4,5). Evidence suggests that the interaction between DDX39A and CIP29 enhances DDX39A’s helicase RNA unwinding activities (32). We hypothesize that during spliceosome formation, DDX39A and DDX39B most likely interact with different factors that can influence their RNA-binding and enzymatic functions.

DDX39A and DDX39B have conserved helicase core domains but differ in the other sequences. It is interesting to note that a single amino acid residue difference in DDX39A and DDX39B (which correspond to C223 and V224, respectively) is responsible for the formation of apo-AREX and apo-TREX complexes by DDX39A and DDX39B, respectively (7). The four-amino-acid motif adjacent to the DECD domain, which is divergent in DDX39A and DDX39B, is necessary and sufficient in dictating the circRNA length preferences of these two paralogs (4). Alphafold-based prediction of DDX39A and DDX39B protein structures indicate that this divergent sequence motif is present on the surface of the protein (36) and may be involved in interacting with RNA or other proteins. DDX39A and DDX39B show the greatest dissimilarity in the first 35 amino acid residues of their N-terminal region. The functional role of this divergent N-terminal region in DDX39 proteins is not yet characterized; however, Shi *et al.* (37) previously showed that a DDX39B mutant lacking the N-terminal region has reduced ATPase activity. Furthermore the N-terminal region in DDX19 negatively regulates its ATPase activity by favoring an open conformation of the core domains in the absence of RNA (38). In fact, it is possible that the divergent N-terminal regions of DDX39A and DDX39B also have an intrinsic autoregulatory function that varies between the two paralogs. Our results from the DDX39A-DDX39B chimera experiments suggest that although important, the N-terminal region is not the only motif responsible for the specific function of DDX39B in RNA splicing. Recently published work from Fujita *et al.*, 2024 (39) also showed that both N- and C-terminal regions of DDX39B are required for its nuclear export complex formation. Mutating the amino acids I411A and S412A in the C-terminal region abrogates DDX39B ability to form the nuclear export complex. The C-terminal domain of DDX39B is also involved in forming high-order complexes with the scaffolding protein SARNP1, and these interactions were found to be necessary for the nuclear export of GC-rich mRNAs (40). We propose that different partners, which interact with the divergent sequence motifs in both the N- and C-terminal halves of DDX39A and DDX39B, determine the unique requirement for DDX39A or DDX39B in RNA splicing.

## Conclusion

This study points out that although DDX39A and DDX39B share similar functions, they play different roles in the splicing of specific RNA substrates. Determining how these paralogs regulate RNA splicing differently and whether these mechanisms are evolutionarily conserved will help us develop a better understanding of the molecular rules that govern the complex regulation of RNA processing in vertebrate species.

## Supplementary Material

gkae431_Supplemental_Files

## Data Availability

There will be no restriction on any material described in this manuscript. The bulk RNA sequencing datasets have been uploaded to the Gene Expression Omnibus with accession number GSE253261. All data are available in the main text or supplementary materials.
