# Supplementary material for: Parsing the roles of DExD-box proteins DDX39A and DDX39B in alternative RNA splicing
Source: Nucleic Acids Res. 2024 May 27;52(14):8534–51. doi: 10.1093/nar/gkae431 (PMC11317157; doi:10.1093/nar/gkae431)
Supplement: gkae431_Supplemental_Files [file gkae431_supplemental_files.zip › Supplementary Figures - combined_v9.pdf]

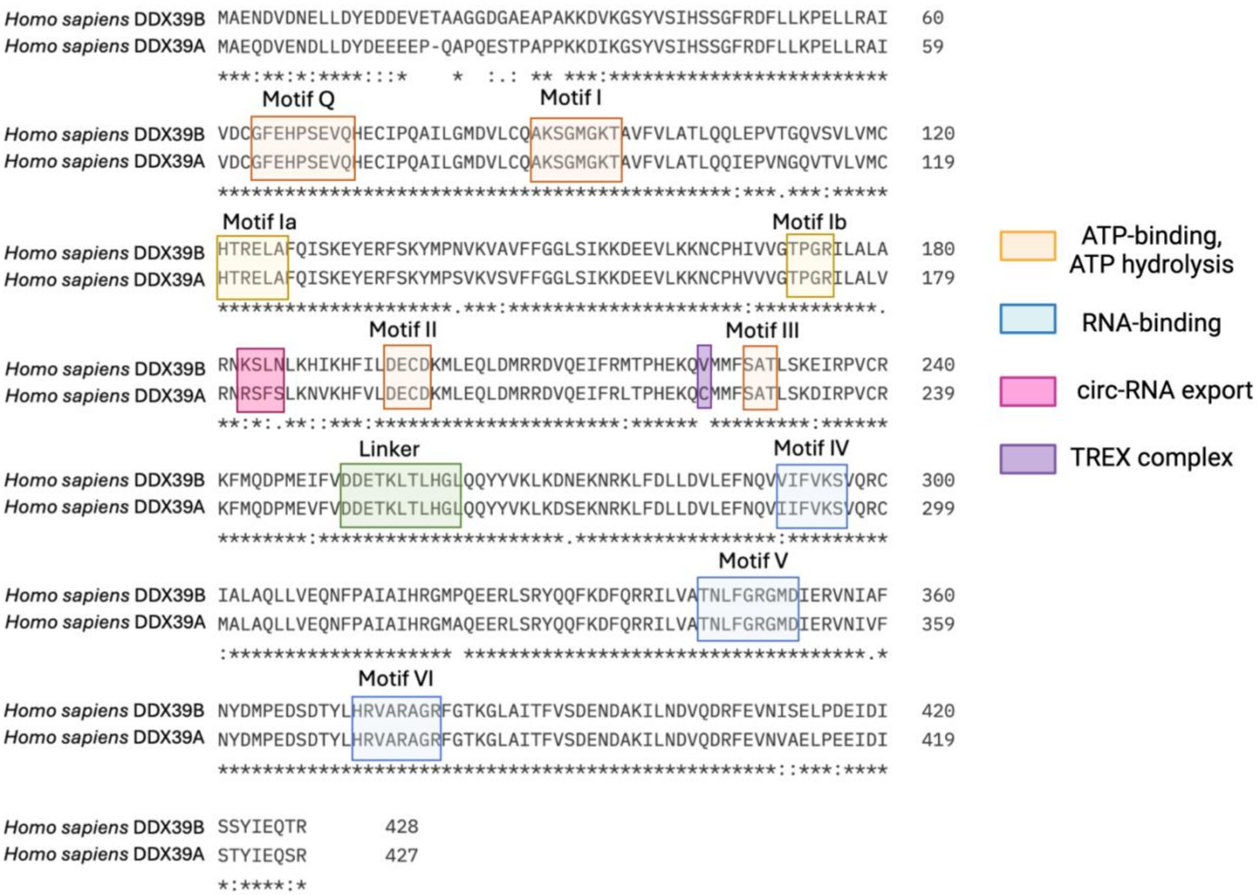

Supplementary Figure 1: Protein alignment of human DDX39A and DDX39B amino acid sequences highlighting the sequence motifs responsible for their functions

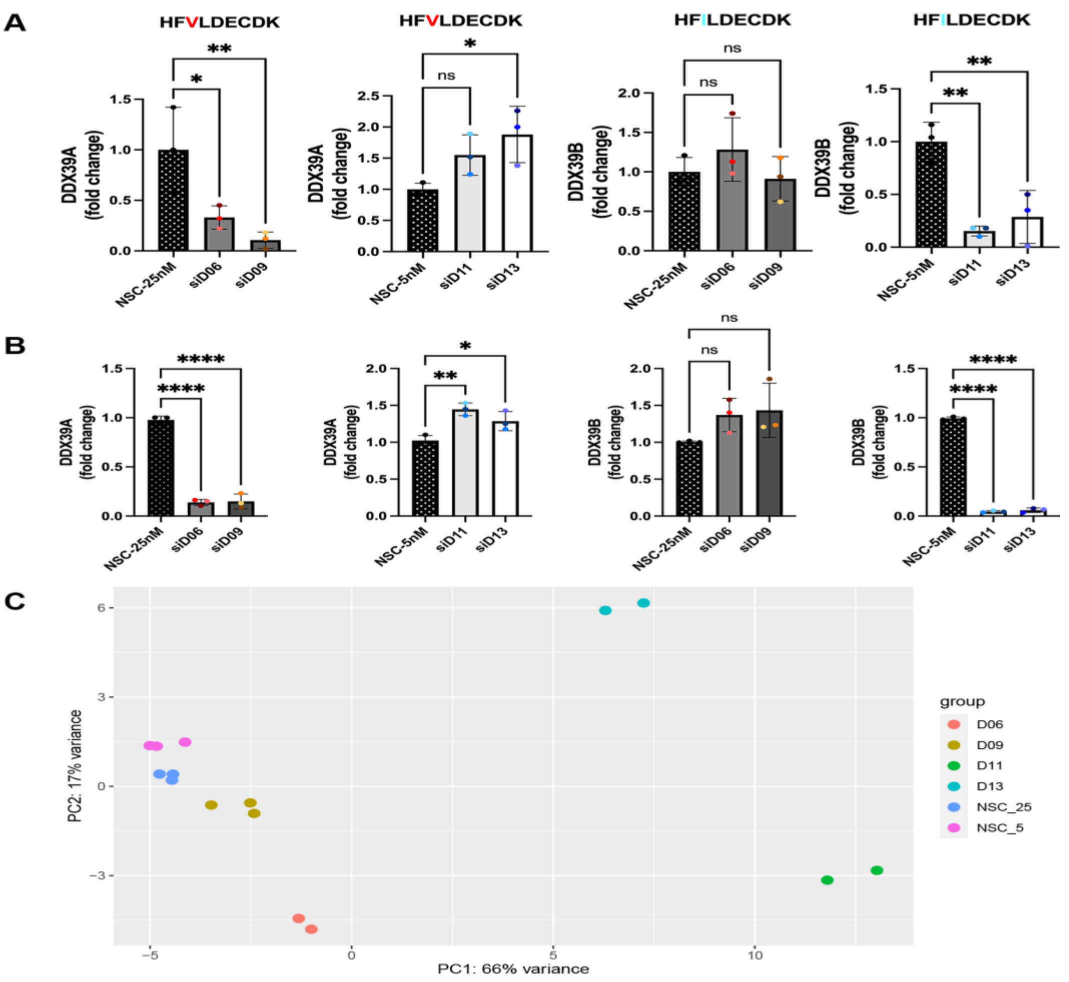

Supplementary Figure 2: A) Levels of DDX39A-specific peptide (HF**V**LDECDK) and DDX39B-specific peptide (HF**I**LDECDK) in lysates from DDX39A-knockdown cells (siD06 and siD09) and DDX39B-knockdown cells (siD11 and siD13) when compared to control cells (NSC-5nM and NSC-25nM). B) Levels of DDX39A and DDX39B transcripts in DDX39A-knockdown and DDX39B-knockdown cells compared to control cells. In all panels, the data are shown as mean ± s.d., and statistical significance was assessed using one-way ANOVA(****$p \leq 0.0001$; **$p \leq 0.01$, *$p \leq 0.05$, ns = not significant). C) Principal component analysis of RNAseq data from DDX39A-knockdown (siD06 and siD09), DDX39B-knockdown (siD11 and siD13), and control (NSC-5nM and NSC-25nM) samples.

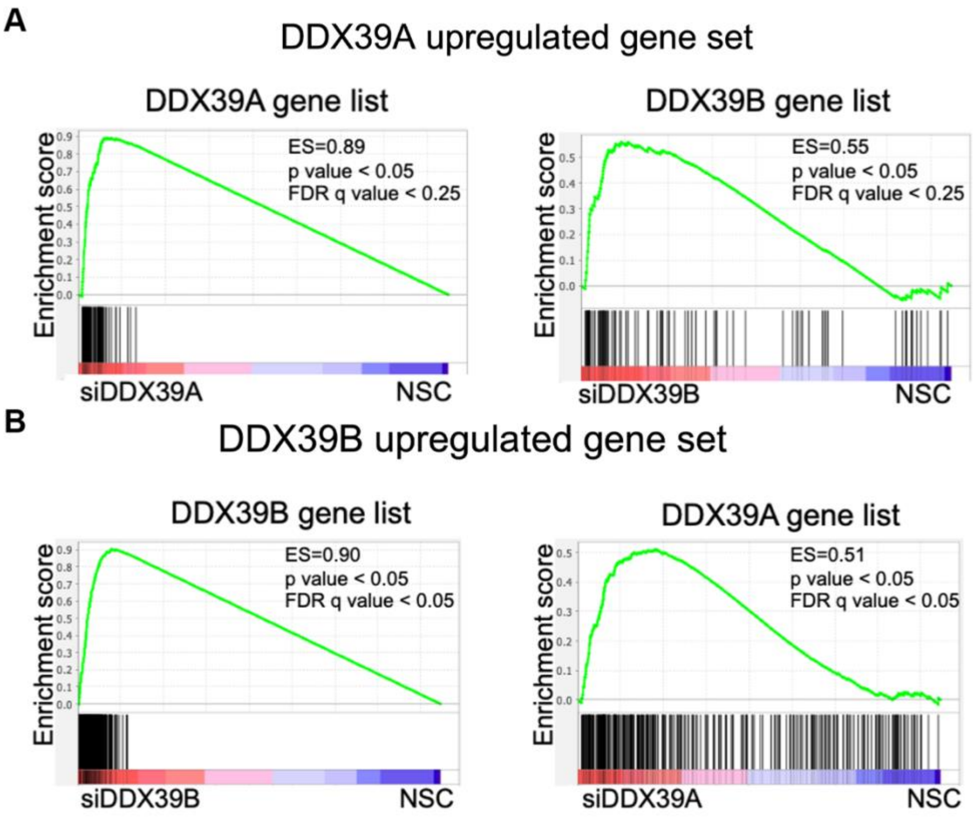

Supplementary Figure 3: A) GSEA results of enrichment of genes upregulated upon DDX39A knockdown in DDX39A-knockdown (left panel) and DDX39B-knockdown (right panel) over control in HeLa cells. B) GSEA results of enrichment of genes upregulated upon and DDX39B knockdown in DDX39B-knockdown (left panel) and DDX39A-knockdown (right panel) over control in HeLa cells.

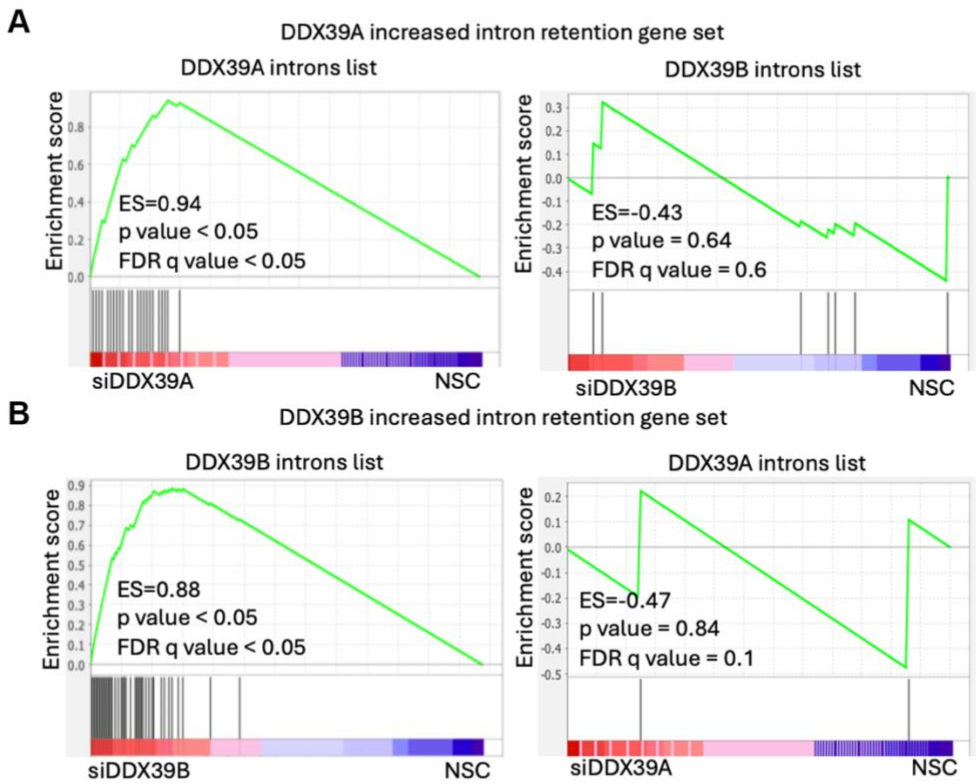

Supplementary Figure 4: GSEA enrichment results of introns retained more upon A) DDX39A knockdown and B) introns retained more upon DDX39B knockdown. Introns retained more upon DDX39A knockdown and DDX39B knockdown are enriched in DDX39A knockdown and DDX39B phenotypes, respectively, compared to controls.

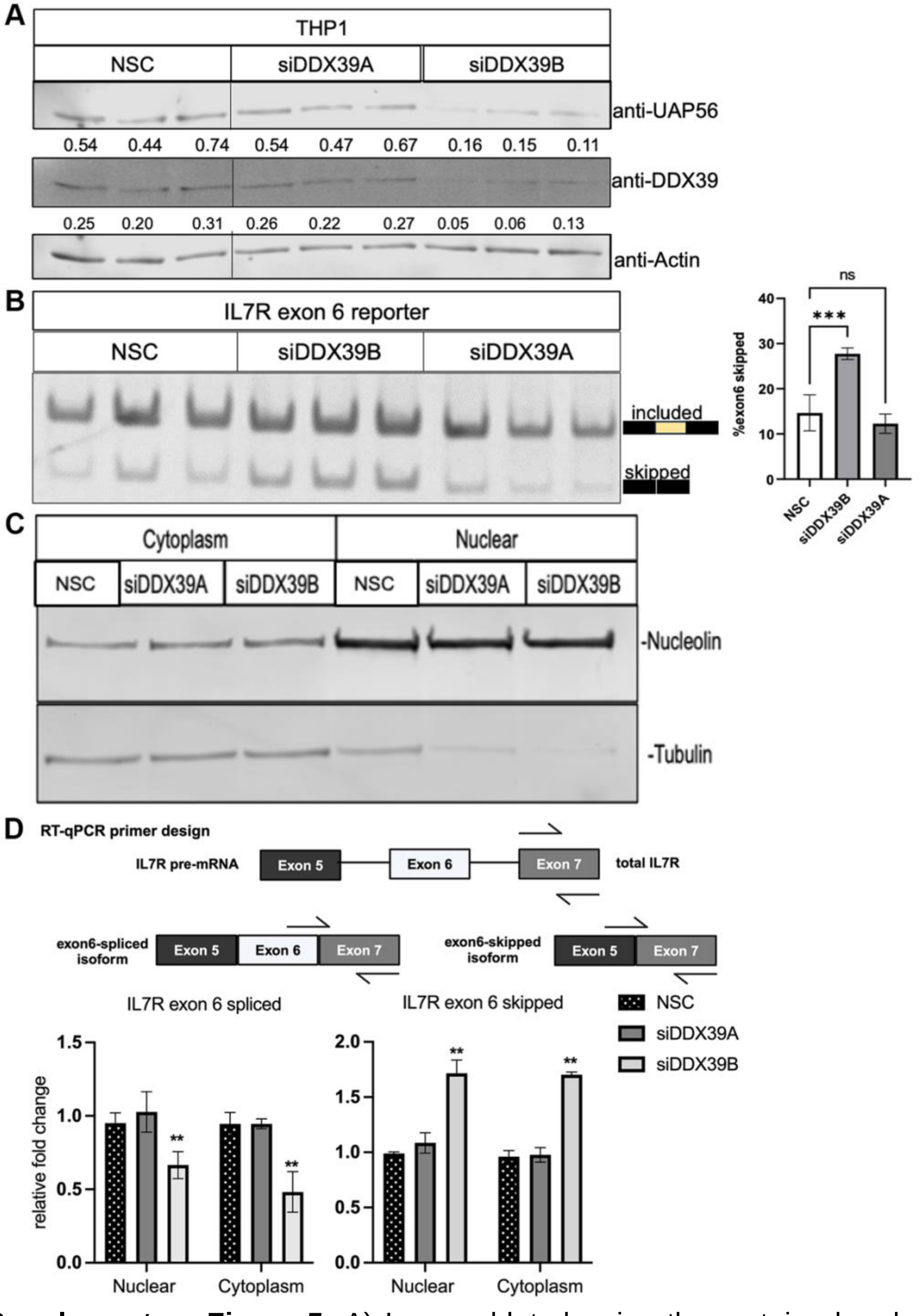

Supplementary Figure 5: A) Immunoblot showing the protein abundance of DDX39B and DDX39A relative to actin in THP1 cells treated with siRNAs targeting DDX39A (siD09) and DDX39B (siD13) expression and non-silencing control (NSC). The numbers indicating protein abundance relative to actin are listed below each immunoblot. It was

difficult to accurately measure DDX39A protein expression because commercially available antibodies targeting DDX39A (anti-DDX39) cross-react with DDX39B. B) RT-PCR analysis of IL7R exon 6 splicing in transcripts from IL7R minigene reporter in HeLa cells. In all panels, the data is shown as mean ± s.d., and statistical significance was assessed using one-way ANOVA (***$p \leq 0.001$; ns = not significant). C) Subcellular fractionation of DDX39A knockdown, DDX39B knockdown, and control HeLa cells. Protein abundances of Nucleolin and Tubulin in different subcellular compartments in control, DDX39A knockdown (siD09) and DDX39B knockdown (siD13) HeLa cells. D) RT-qPCR quantification of the relative abundances of the IL7R exon 6-spliced and IL7R exon6-skipped transcript isoforms in different sub-cellular compartments upon DDX39A and DDX39B knockdown. Statistical significance in transcript isoform abundance in knockdown compared to control was assessed using one-way ANOVA (**$p \leq 0.01$).

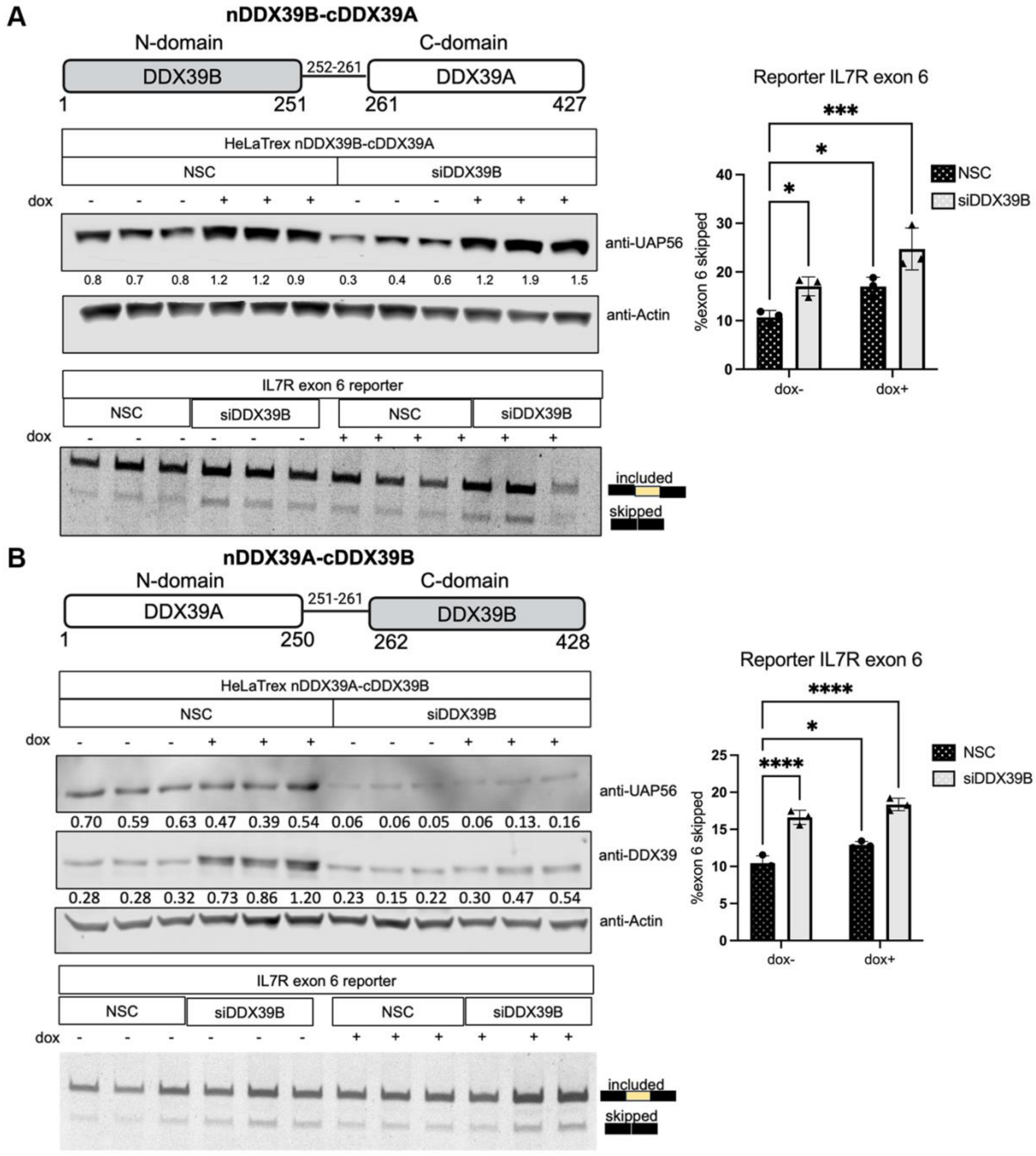

Supplementary Figure 6: DDX39A-DDX39B chimeras cannot rescue IL7R exon 6 splicing upon DDX39B depletion. A) (Top panel) Immunoblot of protein abundance of nDDX39B-cDDX39A overexpression in control and DDX39B-depleted conditions. The numbers indicating protein abundance relative to actin are listed below each immunoblot. (Bottom panel) RT-PCR gel showing no rescue in reporter *IL7R* exon 6 skipping upon

nDDX39B-cDDX39A overexpression. B) (Top panel) Immunoblot of protein abundance of nDDX39A-cDDX39B overexpression in control and DDX39B-depleted conditions. The numbers indicating protein abundance relative to actin are listed below each immunoblot. (Bottom panel) RT-PCR gel showing no rescue in reporter *IL7R* exon 6 skipping upon nDDX39A-cDDX39B overexpression. B) In all panels, the data are shown as mean ± s.d. and statistical significance was calculated using one-way ANOVA (***$p \leq 0.001$; **$p \leq 0.01$; *$p \leq 0.05$).

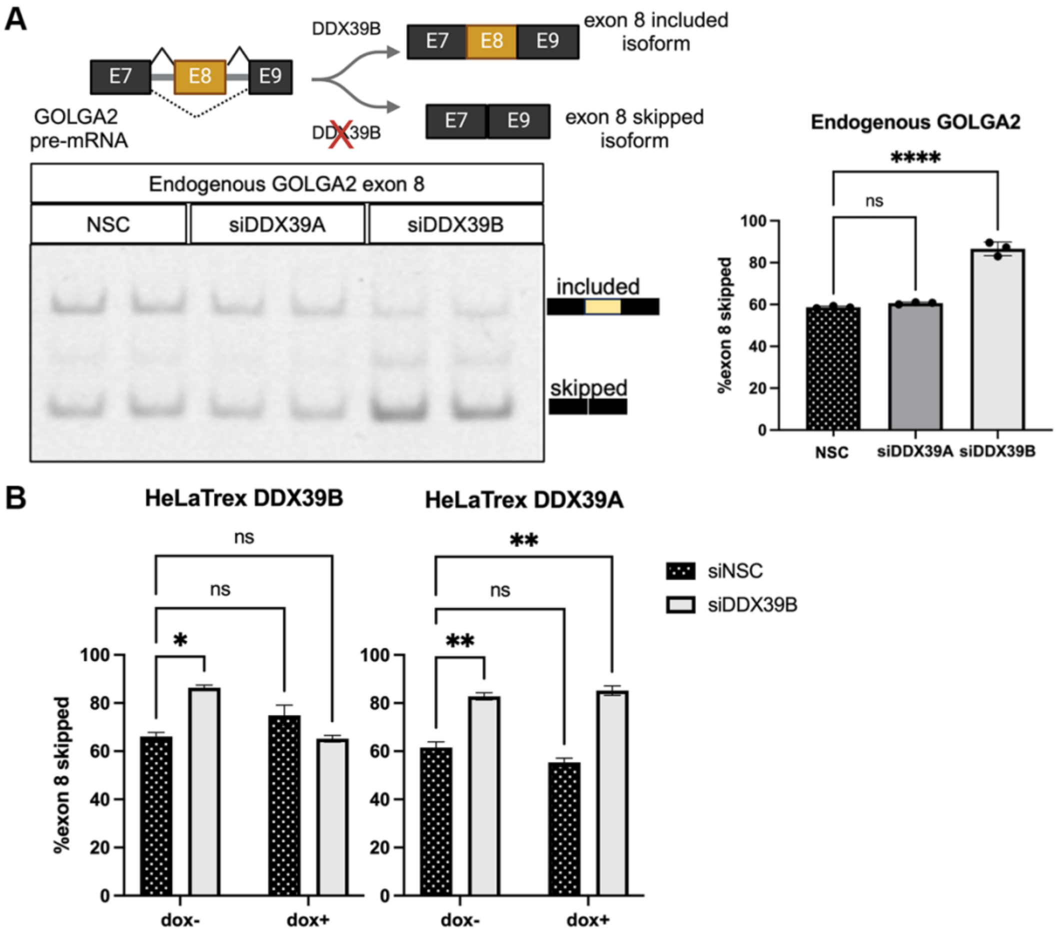

Supplementary Figure 7: A) RT-PCR analysis of exon 8 splicing in transcripts from endogenous *GOLGA2* in DDX39A knockdown (siD09), DDX39B knockdown (siD13) and control (NSC) in HeLa cells. B) Rescue experiments measuring endogenous *GOLGA2* exon 8 splicing using RT-PCR analysis in HeLa cell lines stably expressing DDX39A or DDX39B transgene. In all panels, the data are shown as mean ± s.d., and statistical significance was assessed using one-way ANOVA (**p≤ 0.01; *p ≤ 0.05; ns = not significant).

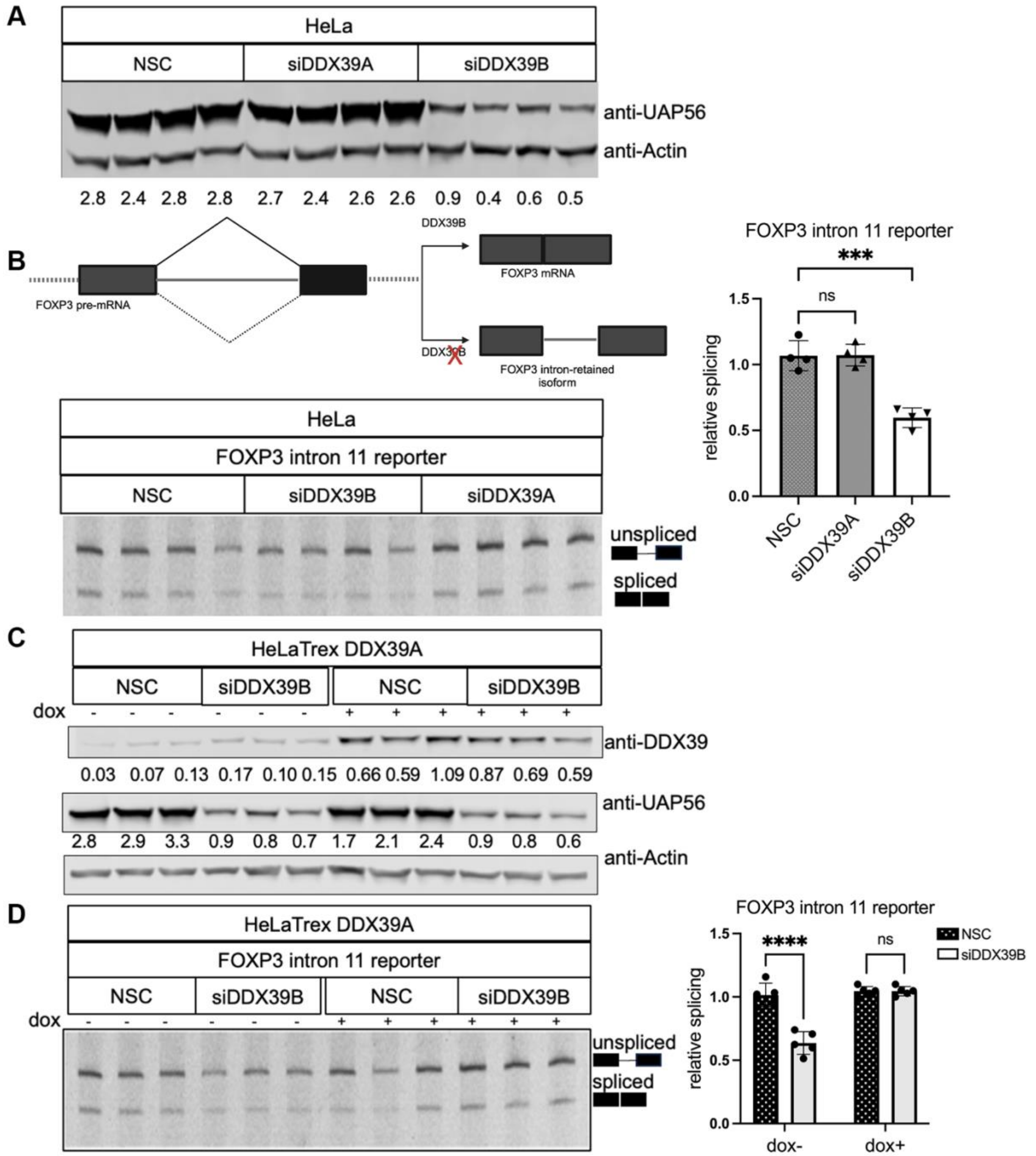

Supplementary Figure 8: Overexpressing DDX39A rescues *FOXP3* intron 11 splicing. *FOXP3* intron 11 splicing in HeLa cells treated with siRNAs siD09 and siD13 targeting DDX39A and DDX39B expression, respectively, and non-silencing control

(NSC). A) Protein abundance of DDX39B relative to actin in Hela cells. The numbers indicating protein abundance relative to actin are listed below each immunoblot. B) The top panel shows a diagrammatic representation of *FOXP3* intron splicing in the presence and absence of DDX39B. Bottom panel shows the RT-PCR analysis of *FOXP3* intron 11 splicing in transcripts from *FOXP3* intron 11 minigene reporter. C-D) Rescue experiments of *FOXP3* intron 11 splicing in HeLa cells stably expressing siRNA-resistant DDX39A transgene. C) The protein abundance of DDX39A and DDX39B relative to actin in HeLa cells stably expressing DDX39A transgene. The numbers indicating protein abundance relative to actin are listed below each immunoblot. D) RT-PCR analysis of *FOXP3* intron 11 splicing in transcripts from minigene reporter. In all panels, the data are shown as mean ± s.d., and statistical significance was calculated using one-way ANOVA (****: $p \leq 0.0001$; **: $p \leq 0.01$; ns = not significant).

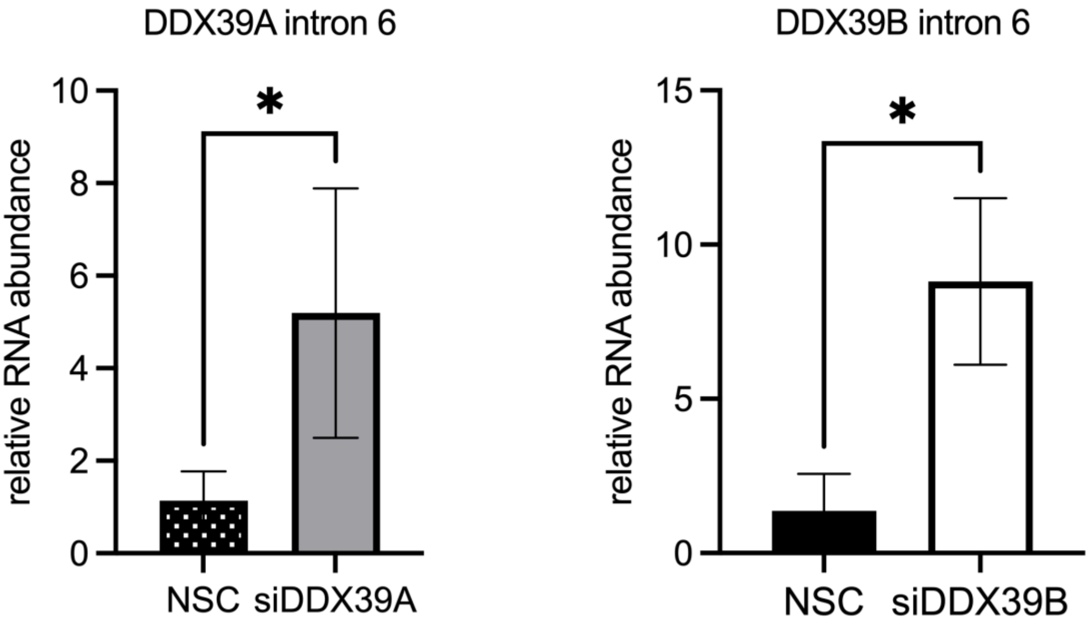

Supplementary Figure 9: DDX39A and DDX39B knockdown results in respective intron retention. RT-qPCR analysis of DDX39A intron 6-retained transcripts relative to total DDX39A transcripts and DDX39B intron 6-retained transcripts relative to total DDX39B transcripts in DDX39A knockdown (siD09), DDX39B knockdown (siD13) and control THP1 cells. In all panels, the data are shown as mean ± s.d., and statistical significance was assessed using a student's t-test (*$p \leq 0.05$).
